# (Phenylamino)pyrimidine-1,2,3-triazole derivatives as analogs of imatinib: searching for novel compounds against chronic myeloid leukemia

**DOI:** 10.3762/bjoc.17.144

**Published:** 2021-09-01

**Authors:** Luiz Claudio Ferreira Pimentel, Lucas Villas Boas Hoelz, Henayle Fernandes Canzian, Frederico Silva Castelo Branco, Andressa Paula de Oliveira, Vinicius Rangel Campos, Floriano Paes Silva Júnior, Rafael Ferreira Dantas, Jackson Antônio Lamounier Camargos Resende, Anna Claudia Cunha, Nubia Boechat, Mônica Macedo Bastos

**Affiliations:** 1 Laboratorio de Sintese de Farmacos – LASFAR, Fundacao Oswaldo Cruz, Instituto de Tecnologia em Farmacos, Farmanguinhos –Manguinhos, CEP 21041-250, Rio de Janeiro, Brazil; 2 Departamento de Química Orgânica, Universidade Federal Fluminense, Campus do Valonguinho, CEP 24020-150,Niterói, Brazil; 3 Laboratório de Bioquímica Experimental e Computacional de Farmacos, Fundaçao Oswaldo Cruz, Instituto Oswaldo Cruz, CEP 21040-900, Rio de Janeiro, Brazil; 4 Instituto de Ciências Exatas e da Terra, Universidade Federal de Mato Grosso, Campus Universitário do Araguaia, CEP 78698-000, Pontal do Araguaia, MT, Brazil

**Keywords:** chronic myeloid leukemia, 1,3-dipolar cycloaddition, imatinib, (phenylamino)pyrimidine-pyridine, 1,2,3-triazole

## Abstract

The enzyme tyrosine kinase BCR-Abl-1 is the main molecular target in the treatment of chronic myeloid leukemia and can be competitively inhibited by tyrosine kinase inhibitors such as imatinib. New potential competitive inhibitors were synthesized using the (phenylamino)pyrimidine-pyridine (PAPP) group as a pharmacophoric fragment, and these compounds were biologically evaluated. The synthesis of twelve new compounds was performed in three steps and assisted by microwave irradiation in a 1,3-dipolar cycloaddition to obtain 1,2,3-triazole derivatives substituted on carbon C-4 of the triazole nucleus. All compounds were evaluated for their inhibitory activities against a chronic myeloid leukemia cell line (K562) that expresses the enzyme tyrosine kinase BCR-Abl-1 and against healthy cells (WSS-1) to observe their selectivity. Three compounds showed promising results, with IC_50_ values between 1.0 and 7.3 μM, and were subjected to molecular docking studies. The results suggest that such compounds can interact at the same binding site as imatinib, probably sharing a competitive inhibition mechanism. One compound showed the greatest interaction affinity for BCR-Abl-1 in the docking studies.

## Introduction

Changes in tyrosine kinase proteins (TKPs), either by mutation or chromosomal translocation, can turn them into potent oncogenes. Continuity and increased signaling are associated with some types of neoplasms, such as chronic myeloid leukemia (CML) [1]. A CML is a neoplasm of the bone marrow that transforms normal hematopoietic progenitor cells into malignant cells [2]. This transformation is marked by the presence of an acrocentric chromosome known as Philadelphia (Ph). Ph is the result of the reciprocal chromosomal conversion between the proto-oncogene *Abl1* of chromosome 9 and the *BCR* gene on chromosome 22 [3–4].

**Figure 1:**
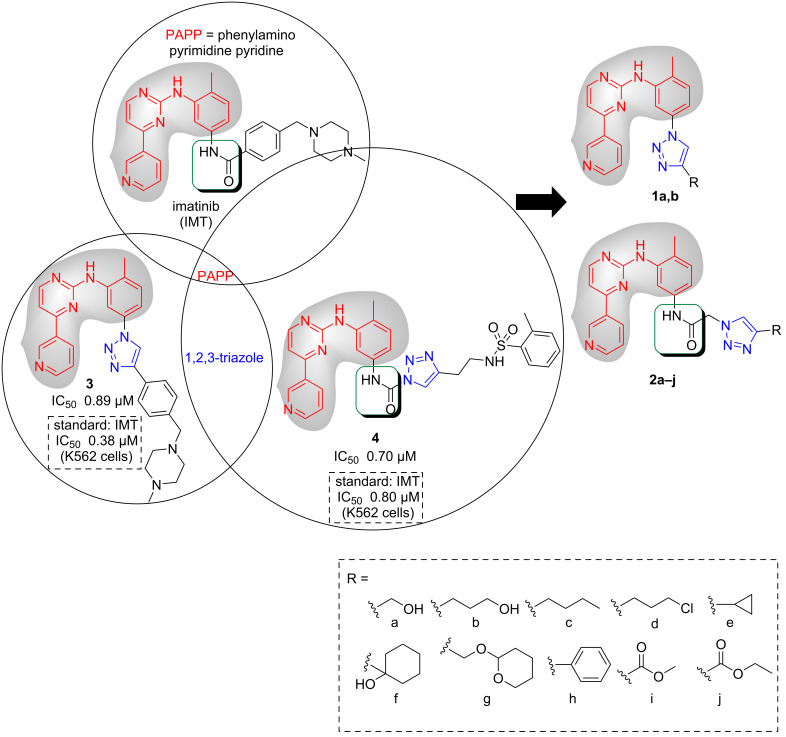
Proposed structural modifications to obtain triazole derivatives **1a**, **b** and **2a**–**j**.

Research activity on compounds able to act as protein tyrosine kinase inhibitors (TKIs), which has intensified since the 1980s, has led to the identification of the (phenylamino)pyrimidine (PAP) structure [5–6]. The addition of an additional pyridine ring to PAP raised its cellular activity, producing PAPP, which, after some more chemical modifications, culminated in imatinib (IMT) [7]. PAPP has been used to develop new TKIs that are even more potent than IMT, such as nilotinib [6–7]. These drugs act as inhibitors at the ATP binding site in the inactive form of BCR-Abl-1, preventing the binding of the protein to ATP in a competitive manner and resulting in the interruption of the substrate phosphorylation process and the transduction of signals; these processes induce cell apoptosis [8–9]. As mutations in the BCR-Abl-1 enzyme domain can occur, cases of resistance have emerged in the treatment with TKIs, compromising its effectiveness [10–11]. In continuation of the work by our group to develop new imatinib analogs [12–13], in this work, we designed a series of imatinib 1,2,3-triazole analogs **1a**,**b** and **2a–j** (Figure 1).

The 1,2,3-triazoles are heterocyclic compounds, consisting of a five-membered ring, containing two carbon atoms and three nitrogen atoms [14]. The application of click chemistry, a concept developed by Sharpless and collaborators, to the Huisgen cycloaddition (a 1,3-dipolar cycloaddition reaction to obtain this heterocycle) allowed the regiospecific synthesis of the 1,4-disubstituted isomers with good yields in the presence of copper(I) salts [15–17]. Since then, medicinal chemists used the 1,2,3-triazole core strategically in the design of new compounds [18–19]. The explanation for this interest is associated with its resistance towards oxidation, reduction, and acidic or basic hydrolysis reactions that occur in phase I of human metabolism [20–21]. The application of this core through isosterism (classical or non-classical) in the design of new active compounds has shown good results [22–23]. Specifically, in the development of new inhibitors for the treatment of cancer [24], its use is associated with producing compounds able to overcoming resistance (primary or secondary) inherent to this type of treatment [25]. In this regard, Arioli and co-workers developed a series of derivatives (from PAPP) containing the 1,2,3-triazole nucleus in place of the amide group (present in the IMT structure) that showed inhibitory activity of recombinant Abl kinase equivalent to the drug of reference [26].

In this context, the intent is to replace the amide group present in IMT by the 1,2,3-triazole nucleus, similar to compound **3** described in the literature by Li and co-workers, which showed a good inhibition profile of the myelogenous leukemia K562 cell line [27].

Considering the good inhibition profile of substance **3** against K562 cells, we proposed, through the molecular simplification strategy, the derivatives **1a** and **1b**, which have hydroxyalkyl groups replacing the phenylpiperazine group. The hydroxymethyl and hydroxypropyl groups were chosen because they were not evaluated in the original work that led to compound **3**, and, additionally, they are able to act as acceptors and donors in hydrogen bonds and thus were expected to increase the aqueous solubility of the compounds.

Additionally, Kalesh and co-workers demonstrated good results with derivative **4** which contains the amide group and the 1,2,3-triazole nucleus [28]. Thus, we designed derivatives **2a–j** comprising both groups, among which we included a methylene spacer in order to increase the degree of conformational freedom and to investigate its impact on the activity and interaction with the enzyme BCR-Abl-1. Regarding the phenylpiperazine group present in compound **3**, we have simplified the side chain attached to the 1,2,3-triazole nucleus by including different groups, such as ester (**2i**,**j**), cycloalkanes (**2e**,**f**), a saturated heterocycle in **2g** and an aromatic ring in **2h** whose stereoelectronic and physicochemical characteristics vary significantly, in order to be able to carry out an initial comparison of the relationship between structure and activity.

## Results and Discussion

### Chemistry

**Scheme 1:**
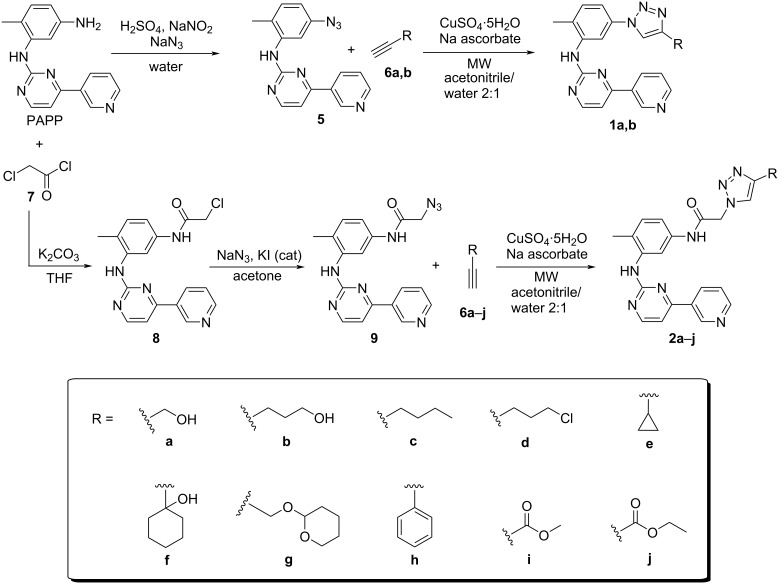
Synthetic route of the triazole derivatives **1a**,**b**, and **2a–j**.

We synthesized twelve 1,4-disubstitued 1,2,3-triazole IMT derivatives **1a**,**b**, and **2a–j** as outlined in Scheme 1.

*N*-(5-Azido-2-methylphenyl)-4-(pyridin-3-yl)pyrimidin-2-amine (**5**) was obtained from the aromatic nucleophilic substitution reaction of intermediate PAPP via the formation of a diazonium salt with 84% yield, which was characterized, and its data agreed with those in the literature [29]. The carbonyl nucleophilic substitution reaction between intermediate PAPP and chloroacetyl chloride (**7**) generated *N*-(4-methyl-3-((4-(pyridin-3-yl)pyrimidin-2-yl)amino)phenyl)chloroacetamide (**8**) in 81% yield, and its characterization data were in accordance with the literature [30]. *N*-(4-Methyl-3-((4-(pyridin-3-yl)pyrimidin-2-yl)amino)phenyl)azidoacetamide (**9**) was obtained from the nucleophilic substitution reaction of intermediate **8** in 85% yield. The ^1^H and ^13^C NMR spectra of compounds **8** and **9** were similar, but in the IR spectrum of intermediate **9**, it was possible to observe the characteristic stretching of the azide group at 2103 cm^−1^.

The 1,3-dipolar cycloaddition reactions via the copper-catalyzed 1,3-dipolar cycloaddition reaction (CuAAC) of the azides **5** and **9** with suitably functionalized acetylenes **6a–j**, using sodium ascorbate and copper sulfate in ACN/H_2_O 2:1 under microwave irradiation were carried out to obtain the 1,4-regioisomers of the final products **1a,b**, and **2a–j**, respectively, with 30–84% yields. This last step was adapted from a method already described in the literature [31].

The formation of compounds **1a,b** and **2a–j** was observed by the disappearance of the characteristic stretching of the azide groups at 2107 and 2103 cm^−1^ in the IR spectra, which are present in intermediates **5** and **9**, respectively, and compounds **2a–j** showed carbonyl absorption at 1671–1660 cm^−1^ (amide). ^1^H NMR spectrum analysis showed the appearance of a single signal at 8.52 ppm and between 8.61–7.79 ppm referring to the hydrogen of the 1,2,3-triazole (C-5) for compounds **1a**,**b** and **2a–j**, respectively. In the ^13^C NMR spectra, the signals in the range of 165.8–163.5 ppm are attributed to the amide carbonyl in products **2a–j**.

The lower yields in the synthesis of the final products **2e–g** can be associated with both the difficulty of purifying some products and the high volatility of some acetylenes used, which have been lost at the time of irradiation.

**Figure 2:**
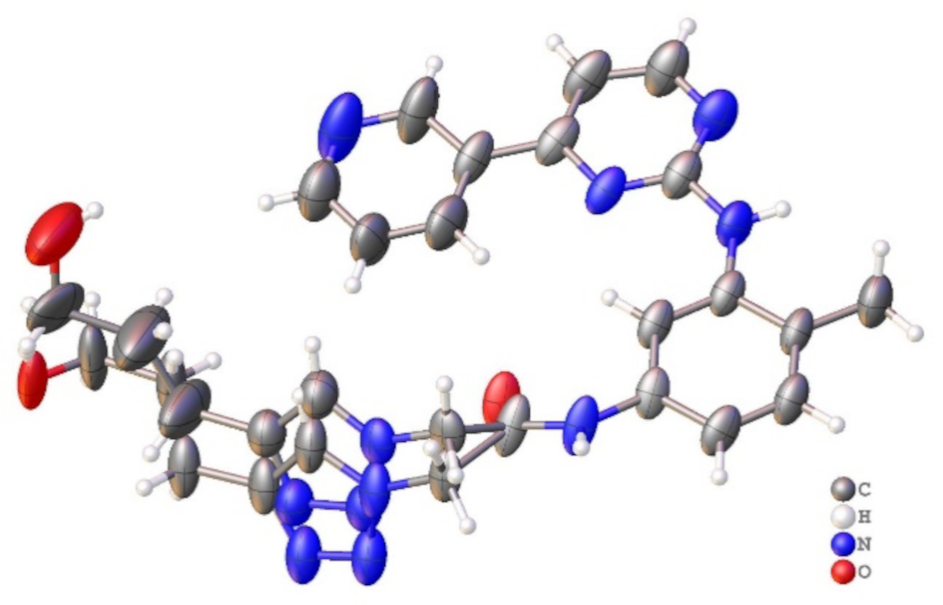
Asymmetric unit representation of the 1,2,3-triazole derivative **2b**. Displacement ellipsoids are drawn at the 50% probability level.

All compounds were obtained with satisfactory purity (>95%) determined by liquid chromatography or elemental analysis. Despite their purity, compounds **2b**, **2d**, **2e**, and **2h** had an absence of some signals in the ^1^H NMR and ^13^C NMR spectra. For structural confirmation of these new derivatives, we also carried out an X-ray diffraction study of compound **2b**. Yellow single crystals suitable for X-ray diffraction analysis were obtained by slow evaporation of a solution of **2b** in dichloromethane. Based on the X-ray crystallographic analysis, the molecular structure was confirmed, as shown in Figure 2.

### Biological assay

**Figure 3:**
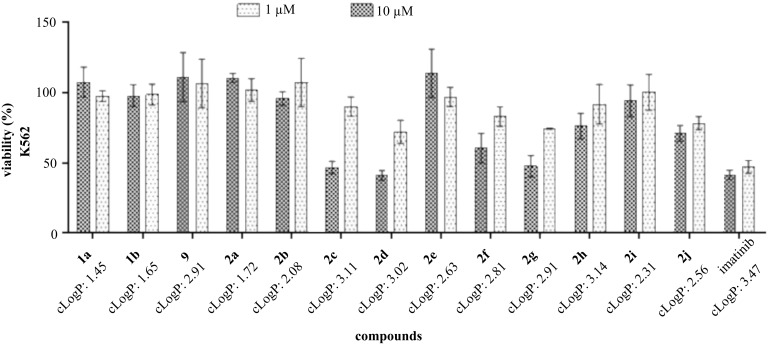
Screening of the triazole derivatives of imatinib **1a**,**b**, and **2a–j** at concentrations of 1 μM and 10 μM against the human K562 cell line. Bars represent the mean ± standard deviation. The standard used was IMT.

The compounds showed significant inhibitory activity at 10 μM but not at 1 μM (Figure 3).

Compounds **2c**, **2d**, and **2g** were the most promising, and concentration–response curves were drawn to determine the cytotoxic concentration (CC_50_) for 50% of both the K562 and WSS-1 cells so that the selectivity indexes (SIs) could be calculated. These data are shown in Table 1.

**Table 1:** Cytotoxic activity, confidence interval, and selectivity index of imatinib and derivatives **2c**, **2d**, and **2g** against K562 and WSS-1 cell lines.

CC_50_ (μM)					selectivity index (SI)^a^

compound	K562	CI^b^	WSS-1	CI	WSS-1/K562

**IMT**	0.08	0.05–0.10	8.9	7.8–10.0	111.2
**2c**	7.3	5.8–9.2	6.6	5.7–7.6	0.9
**2d**	1.0	0.8–1.3	1.4	1.2–1.7	1.4
**2g**	2.3	1.9–2.8	3.9	3.4–4.5	1.7

^a^SI: CC_50_ (WSS-1)/ CC_50_ (cancer cell K562); ^b^CI: confidence interval (95%).

Even with a small number of compounds, the structure–activity relationship (SAR) shows that with the exception of compound **2h**, which presents a phenyl ring as a side chain (R), derivatives with aliphatic side chains showed greater toxic activity against K562 cells (Figure 3). One hypothesis is that the derivatives that present higher lipophilicity (**2c**, **2d**, and **2g**) quantified by the octanol–water partition coefficient (cLogP, calculated by ALOGPS) as shown in Figure 3 [32], may have better passive diffusion through the membrane of the target cell, increasing its concentration in the intracellular medium and consequently its activity.

In relation to CC_50_ values of the compounds selected for the cell viability test (**2c**, **2d**, and **2g**), all were less potent against K562 cells and more toxic to WSS-1 than IMT (used as a positive control), with a low SI (Table 1). According to Xie and co-workers, the high selectivity of IMT is related to an important hydrogen bond between the Glu305 residue and its amide group, which is probably absent when the synthesized compounds interact with Abl domain [33]. It is worth mentioning that compounds with low SIs can act as chemotherapeutics [34–36]. Despite being less potent than IMT, derivatives **2c**, **2d**, and **2g** were capable of inhibiting K562 cells on a micromolar scale. Thus, these compounds can be used as a prototype for designing new series of substances with greater potency and less toxicity than IMT, with lesser effects for the patient.

### Molecular docking

Validation of the molecular docking protocol was performed through redocking of the IMT complexed to the BCR-Abl-1 structure (PDB code: 3PYY) [37]. Thus, the predicted mode with the lowest energy presented a MolDock value of −206.022 arbitrary units (a.u.) and a mean-square deviation of 1.68 Å, validating the molecular docking protocol with RMSD values below 2.00 Å [38].

The results, using the validated molecular docking protocol, show that **2c**, **2d**, and **2g** interact with BCR-Abl-1 at the same binding site as IMT but show differences in the binding modes and with higher values of interaction energy. Compound **2c** presented a MolDock value of −152.993 a.u. For compound **2d**, the value was −152.127 a.u., and for compound **2g**, it was −167.520 a.u. (Table 2).

**Table 2:** Summary of the interactions of each inhibitor and imatinib with the tyrosine kinase BCR-Abl-1 model.

inhibitors	H-bond energy (a.u.)^a^	residues (H-bond interaction)	steric interaction energy by PLP (a.u.)^b^	residues (steric interactions)	MolDock score (a.u.)

**IMT**	−8.97	Asp400, Glu305, Met337, Thr334	−193.66	Asp400, Glu305, Ile379, Met337, Thr334	−206.02
**2c**	−1.31	Asp400, Leu373	−151.68	Asp400, Glu301, Glu305, Ile379, Leu373, Met309, Val308	−152.99
**2d**	−2.19	Asp400, His380, Ile379	−149.93	Ala399, Asp400, Glu305, His380, Ile312, Leu373, Met309, Phe378, Val318, Val398	−152.13
**2g**	−2.62	Asp400	−164.90	Ala399, Asp400, Glu305, His380, Ile312, Ile379, Leu317, Lys290	−167.52

^a^Arbitrary units. ^b^Piece wise linear potential.

**Figure 4:**
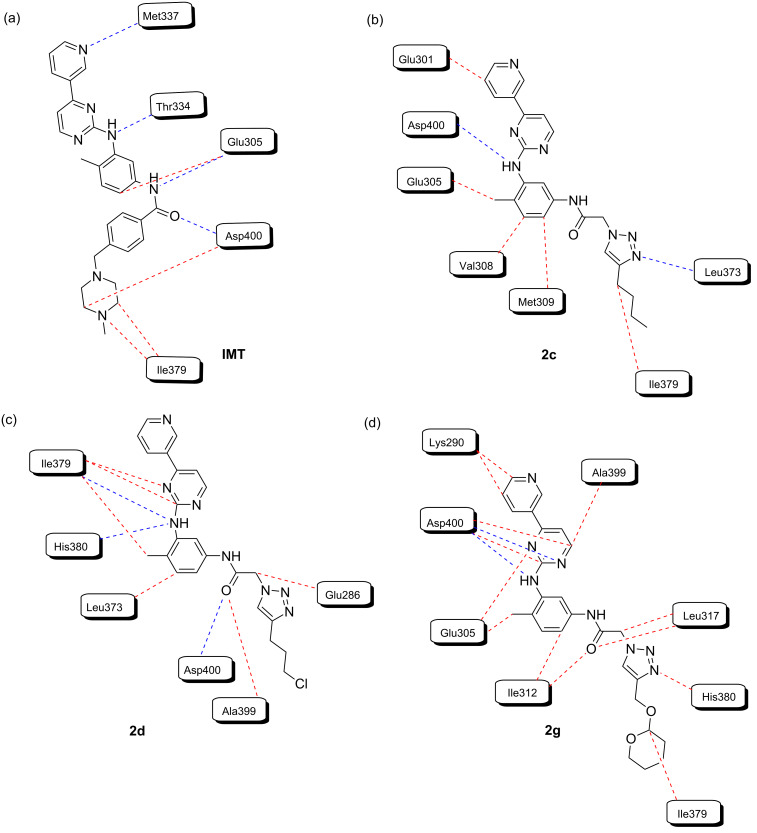
Interaction maps of IMT, **2c**, **2d**, and **2g** with the BCR-Abl-1 structure (PDB code: 3PYY), showing steric interactions (red dotted lines) and hydrogen bonds (blue dotted lines).

Additionally, the analysis of the interactions between IMT and the enzyme BCR-Abl-1 shows hydrogen bonds with Glu305, Thr334, Met337, and Asp400 (hydrogen bonding energy = −8.97 a.u.) and steric interactions with Glu305, Thr334, Met337, Ile379, and Asp400 (steric binding energy = −193,658 a.u.), in agreement with the interactions found for IMT in the 3PYY crystal. The analysis of the inhibitor complexes **2c** and **2g** and BCR-Abl-1 showed interactions similar to those described for IMT (Figure 4a). Thus, compound **2c** presented hydrogen-bonding interactions with Asp400 and Leu383 (hydrogen bonding energy of −1.31 a.u.) and steric interactions with Glu301, Glu305, Val308, Met309, Leu373, Ile379, and Asp400 (steric interaction energy = −151.683 a.u.) (Figure 4b). Similarly, compound **2g** showed a hydrogen-bonding interaction with Asp400 (hydrogen bonding energy = −2.62 a.u.) and steric interactions with Lys290, Glu305, Ile312, Leu317, Ile379, His380, Ala399, and Asp400 (steric interaction energy = −164,897 a.u.) (Figure 4d), presenting the best overlap with the co-crystalized IMT structure. However, compound **2d** showed hydrogen-bonding interactions with Asp400, His380 and Ile379 (hydrogen bonding energy = −2.19 a.u.) and steric interactions with Glu305, Met309, Ile312, Val318, Leu373, Phe378, His380, Val398, Ala399, and Asp400 (steric interaction energy = −149,932 a.u.) (Figure 4c).

Therefore, the computational simulations suggest that the synthesized compounds interact at the same binding site as IMT, probably sharing a competitive inhibition mechanism. The predicted affinity between IMT and this enzyme was higher than the affinities found for compounds **2c**, **2d**, and **2g**. Among the newly synthesized derivatives, compound **2g** presented the greatest interaction affinity for BCR-Abl-1 when compared to compounds **2c** and **2d**, which presented almost equivalent interaction energy values.

In addition, the hydrogen bond between the amino group of IMT with the Thr334 residue [32,38] (Figure 4a) was not observed for compounds **2c**, **2d**, and **2g**, which suggests a lower binding energy of the synthesized compounds when compared to IMT (Table 2). The lacking of that interaction prevents the pyridine ring from mimicking the ATP adenine in the hinge region [39]. As previously mentioned, the absence of the hydrogen-bond interaction between the Glu305 residue and the amide group of **2c**, **2d**, and **2g** (Figure 4b–d) also corroborates their low SI (Table 1).

## Conclusion

A new series of 1,2,3-triazole derivatives of IMT that contain the main pharmacophoric group PAPP has been synthesized and characterized. All compounds **1a,b**, and **2a–j** were evaluated in an in vitro cell test; these compounds included intermediate **9** and IMT (reference drug). Three of the compounds, **2c**, **2d**, and **2g**, were found to be active, with IC_50_ values between 1.0 and 7.3 μM, while the IC_50_ of the reference compound was 0.08 μM. Molecular docking studies suggested that the synthesized compounds **2c**, **2d**, and **2g** interact at the same binding site as IMT, probably sharing a competitive inhibition mechanism. In addition, among the three synthesized derivatives, compound **2g** had the highest interaction affinity for BCR-Abl-1 when compared to compounds **2c** and **2d**, which presented almost equivalent interaction energy values. However, these results cannot be directly correlated with in vitro antimyeloproliferative assessments, which require an enzyme inhibition assay against BCR-Abl-1.

## Experimental

The experimental details and analytical data for intermediates **5**, **8**, and **9** and the title compounds **1a**,**b**, and **2a–j** are given in Supporting Information File 1. The chemical structures of the title compounds were confirmed by ^1^H and ^13^C NMR spectroscopic analyses and HRMS spectrometric analyses.

### X-ray data collection and structure refinement

Single crystal X-ray data for **2b** were collected on a Bruker D8 Venture diffractometer using graphite-monochromated Mo Kα radiation (λ = 0.71073 Å) at 298 K. Data collection, cell refinement, and data reduction were performed with Bruker Instrument Service v4.2.2, APEX2 [40] and SAINT [41], respectively. Absorption correction using equivalent reflections was performed with the SADABS program [35]. The structure solutions and full-matrix least-squares refinements based on *F*^2^ were performed with the SHELX package [42–44] and were refined with fixed individual displacement parameters [Uiso\(H) = 1.2 Ueq (Csp^2^ and C_ar_) or 1.5 U_eq_ (Csp^3^)] using a riding model. All nonhydrogen atoms were refined anisotropically. Some crystallization and X-ray diffraction experiments for compound **2b** were performed. All samples evaluated had low scattering patterns and germinated crystals. This resulted in poor quality data, which limited the quality of the refinement. Structure illustrations were generated using ORTEP-3 for Mercury [45], and crystallographic tables were constructed using Olex2 [46]. X-ray crystallographic data in cif format are available at CCDC 2073131 can be obtained free of charge via http://www.ccdc.cam.ac.uk/data_request/cif.

### Crystal Data of **2b**

C_23_H_24_N_8_O_2_ (*M* = 444.50 g/mol): monoclinic, space group *P*2_1_/*c*, *a* = 14.895(3) Å, *b* = 16.167(3) Å, *c* = 9.5274(14) Å, β = 92.150(5)°, *V* = 2292.7(7) Å^3^, *Z* = 4, *T* = 298.0 K, μ(Mo Kα) = 0.087 mm^−1^, F (000) = 936.0, crystal size = 0.24 × 0.22 × 0.096 mm^3^, *D*_calc_ = 1.288 g/cm^3^; of the 21431 reflections measured (3.72° ≤ 2Θ ≤ 50.682°), 4199 were unique (*R*_int_ = 0.1422, R_sigma_ = 0.0980) and were used in all calculations. The final *R*_1_ was 0.1284 (I >2σ(I)), and *wR*_2_ was 0.3858 (all data).

### Cell line and cell culture

All novel compounds synthesized (**1a**,**b**, and **2a–j**) were evaluated for their activity against K562 cells (ATCC^®^ CRL-243TM), a CML cell line, and a WSS-1 healthy cell line (ATCC^®^ CRL-2029TM). The K562 strain was grown in RPMI-1640 medium according to the provider's recommendations [47–48]. Cultivation of WSS-1 cells was performed in DMEM high glucose medium (Vitrocell) according to ATCC recommendations. The media were supplemented with 10% fetal bovine serum (FBS) and 50 mg/MI of the antibiotic gentamicin, and all cell lines were grown in a cell culture bottle, which had a 0.22 µm pore membrane filter on the lid, allowing the circulation of CO_2_. WSS-1 cells grew adherently, while cells of the K562 strain grew in suspension. All strains were maintained at 35 °C, at 5% CO_2_.

### Cell viability assay

WSS-1 cells were incubated at 5 × 10^4^ (cells per well) in black plates with a 96-well transparent bottom (Greiner Bio-One) for approximately 20 hours to allow for cell growth and adhesion. The compounds obtained were added at concentrations of 1 µM and 10 µM in DMSO and incubated for 46 hours. This assay was performed in triplicate and measured using resazurin (Sigma-Aldrich) at a concentration of 0.1 mg/mL using a FlexStation 3 microplate reader (Molecular Devices).

### K562 cells assay

After plating (2 × 10^4^ cell/mL), the K562 cells were incubated for one hour, and then the compounds dissolved in DMSO (Sigma-Aldrich) were added and further incubated for 47 hours. After this period, resazurin was added at a final concentration per well of 0.01 mg/mL, and the first fluorescence reading was immediately performed (λ_ex_ = 560 nm; λ_ex_ = 590 nm) (time zero) using FlexStation 3 microplates (Molecular Devices). After the first reading, the plate was returned to the incubator, and after one hour, the second reading was performed, completing the 48 hours of incubation with the compounds. The reference inhibitor used was IMT at fixed concentrations of 1 μM and 10 μM.

### Molecular docking

The Molegro Virtual Docker v6.0 (MVD) program was used to predict the complexes and energies of interaction between the enzyme BCR-Abl 1 and inhibitors **2c**, **2d**, and **2g**. The crystallographic structures of the enzyme BCR-Abl-1, complexed with IMT, were extracted from the Protein Data Bank (PDB code: 3PYY) [37]. The structures of the compounds were constructed and optimized by the semi-empirical Recife Model 1 (RM1) method using the Spartan 14 program (Wavefunction, Inc.).

Validation of the molecular docking protocol was performed through the redocking of IMT complexed with the BCR-Abl-1 structure (PDB code: 3PYY), free of water molecules and cofactors, using Molegro Virtual Docker 6.0 (MVD) [49]. Thus, the whole enzyme was set as the center of the search space. Additionally, the search algorithm MolDock optimizer was used with a minimum of 200 runs, and the parameter settings were as follows: population size = 300; maximum iteration = 2000; scaling factor = 0.50; offspring scheme = Scheme 1; termination scheme = variance-based; crossover rate = 0.90. Due to the stochastic nature of the algorithm search, ten independent simulations per ligand were performed to predict the binding mode. Consequently, the complexes with the lowest interaction energy were evaluated. The interactions between BCR-Abl-1 and each inhibitor were analyzed using the ligand map algorithm, a standard algorithm in the MVD program [50]. The usual threshold values for H-bonds and steric interactions were used. All figures for molecular docking results were edited using the Visual Molecular Dynamics 1.9.3 (VMD) program (available for download at http://www.ks.uiuc.edu/Research/vmd/vmd-1.9.3/).

## Supporting Information

File 1 Additional experimental and analytical data, and NMR spectra of synthesized compounds.
