# Supplementary material for: (Phenylamino)pyrimidine-1,2,3-triazole derivatives as analogs of imatinib: searching for novel compounds against chronic myeloid leukemia
Source: Beilstein J Org Chem. 2021 Sep 1;17:2260–9. doi: 10.3762/bjoc.17.144 (PMC8450943; doi:10.3762/bjoc.17.144)
Supplement: File 1 — Additional experimental and analytical data, and NMR spectra of synthesized compounds. [file Beilstein_J_Org_Chem-17-2260-s001.pdf]

BEILSTEIN JOURNAL OF ORGANIC CHEMISTRY

Supporting Information

for

(Phenylamino)pyrimidine-1,2,3-triazole derivatives as analogs of imatinib: searching for novel compounds against chronic myeloid leukemia

Luiz Claudio Ferreira Pimentel, Lucas Villas Boas Hoelz, Henayle Fernandes Canzian, Frederico Silva Castelo Branco, Andressa Paula de Oliveira, Vinicius Rangel Campos, Floriano Paes Silva Júnior, Rafael Ferreira Dantas,
Jackson Antônio Lamounier Camargos Resende, Anna Claudia Cunha, Nubia Boechat and Mônica Macedo Bastos

Beilstein J. Org. Chem. **2021,** *17,* 2260–2269. doi:10.3762/bjoc.17.144

Additional experimental and analytical data, and NMR spectra of synthesized compounds

License and Terms: This is a supporting information file under the terms of the Creative Commons Attribution License (https://creativecommons.org/licenses/by/4.0). Please note that the reuse, redistribution and reproduction in particular requires that the author(s) and source are credited and that individual graphics may be subject to special legal provisions.
The license is subject to the *Beilstein Journal of Organic Chemistry* terms and conditions: (https://www.beilstein-journals.org/bjoc/terms)

Table of contents

1. General information.. S2
2. Procedure for intermediate **5**.. S3
3. Procedure for intermediate **8**.. S3
4. Procedure for intermediate **9**.. S4
5. General procedure for the synthesis of compounds **1a**, **b**, and **2a–j**... S6
6. ^{1}H NMR, ^{13}C NMR, HRMS spectrometric data and purity by HPLC for compounds **1a**, **b**, and **2a–j**...................................... S7–S36
7. Concentration–response curves of imatinib and compounds **2c**, **2d**, and **2g** (WSS-1 and K562). S37
8. References... S38

General information

The reagents were acquired from Sigma-Aldrich and used without prior purification. The solvents used were purchased from Tedia and Vetec. The chromatoplates used were SiliCycle 60F254 plates with an indicator in the ultraviolet (UV) region (254 nm). The melting points were determined in a BüchiB-545 apparatus, and the values were not corrected. A CEM Discover Microwave Synthesizer microwave oven (CEM Corporation, NC, USA) was used in microwave-assisted reactions. The infrared spectra (IR) were obtained on a Thermo Scientific spectrophotometer, model Nicolet 6700. Nuclear magnetic resonance (NMR) spectra were determined on a Bruker HC spectrometer at 400.00 MHz for hydrogen and 100.00 MHz for carbon. Trimethylsilane (TMS) was used as an internal reference standard for hydrogen and carbon (0 ppm). High-resolution mass spectrometry (HRMS) spectra were obtained on a Maxis 3G mass spectrometer with an electrospray ionization source (ESI-MS). The purity of the compounds was determined by high-performance liquid chromatography (HPLC) with a UV detector for elemental analysis. For elemental analysis, a Perkin Elmer 2400 Series II elementary analyzer was used.

Procedure for intermediate 5

***N*-(5-Azido-2-methylphenyl)-4-(pyridin-3-yl)pyrimidin-2-amine (5)**

2-((5-Amino-2-methylphenyl)amino)-4-pyridin-3-yl-pyrimidine ((phenylamino)pyrimidine pyridine, **PAPP**, 3.62 mmol, 1.0036 g), 40 mL of distilled water and concentrated H_2SO_4 (3 mL) were stirred in an ice bath until complete solubilization. Then, $NaNO_2$ (5.42 mmol, 0.3791 g, 1.5 equiv) dissolved in 2 mL of ice-cold water and, subsequently, NaN_3 (7.24 mmol, 0.4704 g, 2 equiv) dissolved in 5 mL of distilled water were added. The reaction was protected from light, stirred for 3 hours at room temperature, and monitored by TLC using a mixture of chloroform/methanol 9:1 as the eluent. After the end of the reaction, the mixture was neutralized with solid K_2CO_3 to pH 7–8. The mixture was filtered, and the solution was concentrated under reduced pressure, obtaining PAPP azide **5** after drying, which was stored in an amber flask [1]. The obtained substance was a yellow solid. Yield: 84%. The analytical data corresponded to the literature [2,3].

Procedure for intermediate 8

***N*-(4-Methyl-3-((4-(pyridin-3-yl)pyrimidin-2-yl)amino)phenyl)chloro-acetamide (8)**

PAPP (1.80 mmol, 0.50 g), anhydrous K_2CO_3 (4.71 mmol, 0.65 g, 2.5 equiv) and 15 mL of anhydrous THF were added to a flask. Then, a solution containing chloroacetyl chloride (**7**, 0.3 mL, 1.1 equiv) dissolved in 15 mL of dry THF was added slowly using a pressure-equalized funnel. The reaction was kept for 1 hour at room temperature and the progress monitored by TLC using a mixture of chloroform/methanol 95:5 as the eluent. Afterwards, 20 mL of distilled water were

added, and a yellow precipitate formed, that was filtered off, and washed with distilled water. Yellow solid; yield: 81%. The analytical data corresponded to the literature [4].

Procedure for intermediate 9

***N*-(4-Methyl-3-((4-(pyridin-3-yl)pyrimidin-2-yl)amino)phenyl)azido-acetamide**

Under an inert atmosphere (N_2), **8** (0.50 mmol, 0.176 g), NaN_3 (0.75 mmol (0.0485 g, 1.5 equiv), KI (0.10 mmol, 0.0163 g, 20 mol %), and 15 mL of anhydrous acetone were added to a flask. The reaction mixture was refluxed for 4 hours and monitored by TLC using chloroform as the eluent. After the removal of the solvent, the solid was washed with water and filtered, yielding an orange solid, which was stored in an amber flask.

Orange solid; yield: 85%, m.p.:187-188 °C. IR (cm^{-1}; film): 2103 (N=N=N str.). Anal. Calcd. (%) for CHN: C, 59.99; H, 4.48; N, 31.09. Found (%): C, 59.71; H, 4.49; N, 31.24. HR-MS (ESI) *m/z* calculated for $C_{18}H_{16}N_8O$ $[M+Na]^+$: 383.1345. Found *m/z* 383.1334 $[M+Na]^+$.

^1H NMR (DMSO-d_6, 400 MHz): 2.21 (s, 3H, CH_3), 4.03 (s, 1H, CH_2), 7.18 (d, 1H, *J*= 8.3 Hz, Ar-H), 7.29 (dd, 1H, *J*= 8.2, 2.1 Hz, Ar-H), 7.44 (d, 1H, *J*= 5.2 Hz, H-pyrimidine), 7.50-7.57 (m, 1H, H-pyridine), 7.91 (d, 1H, *J*= 1.9 Hz, Ar-H), 8.48 (dt, 1H, *J* = 8.0, 1.9 Hz, H-pyridine), 8.51 (d, 1H, *J* = 5.1 Hz, H-pyrimidine), 8.70 (dd, 1H, *J*= 4.8, 1.5 Hz, H-pyridine), 8.95 (s, 1H, NH), 9.26 (d, 1H, *J*= 1.8 Hz, H-pyridine), 10.10 (s, 1H, NH). ^{13}C NMR (DMSO-d_6, 101 MHz): 17.00, 50.66, 107.06, 114.91, 115.31, 123.22, 126.80, 129.70, 131.60, 133.85, 135.78, 137.37, 147.55, 150.79, 158.87, 160.45, 161.03, 165.45.

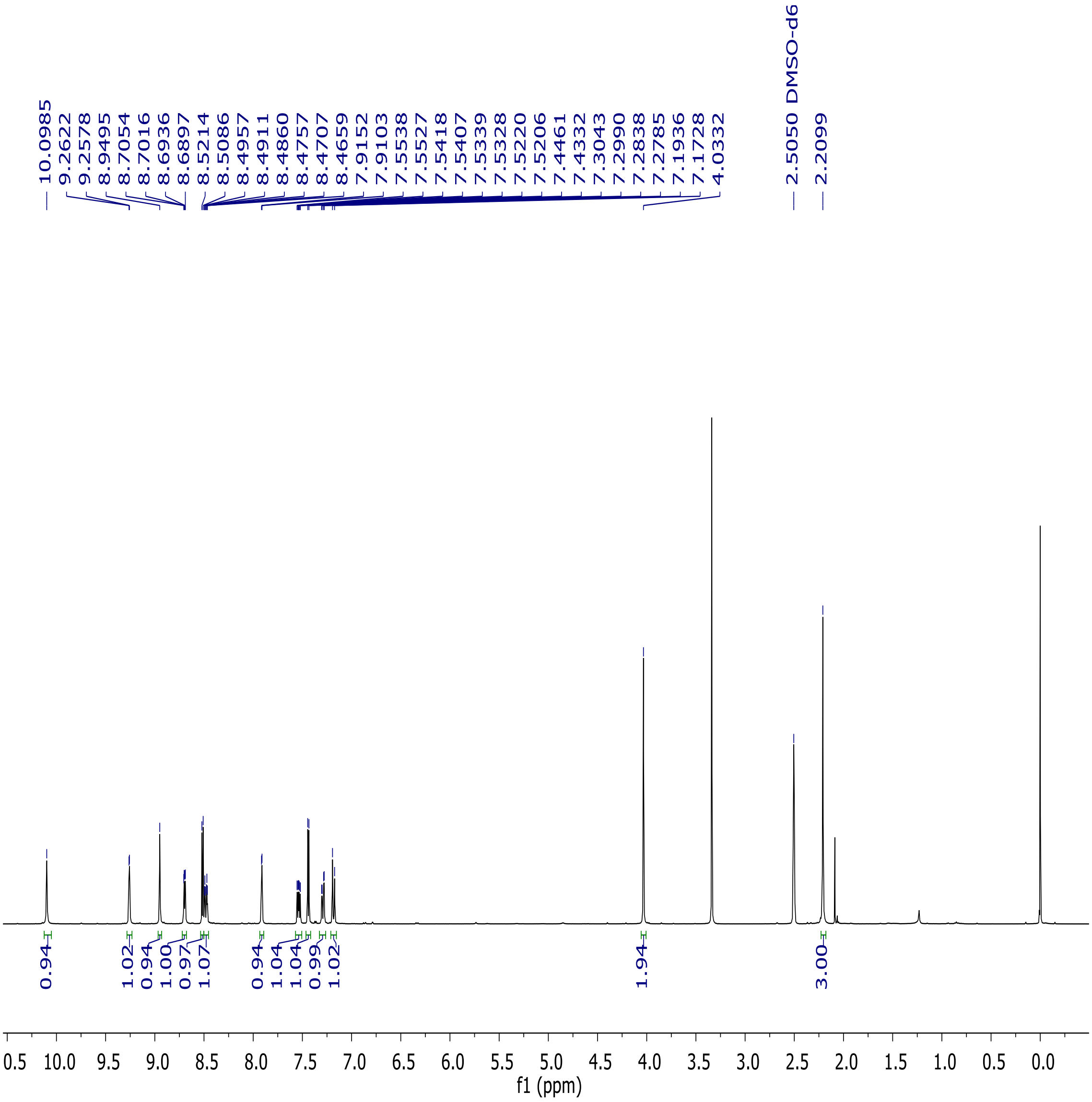
10.0985
9.2622
9.2578
8.9495
8.7054
8.7016
8.6936
8.6897
8.5214
8.5086
8.4957
8.4911
8.4860
8.4757
8.4707
8.4659
7.9152
7.9103
7.5538
7.5527
7.5418
7.5407
7.5339
7.5328
7.5220
7.5206
7.4461
7.4332
7.3043
7.2990
7.2838
7.2785
7.1936
7.1728
4.0332
2.5050 DMSO-d6
2.2099
0.94
1.02
0.94
1.00
0.97
1.07
0.94
1.04
1.04
0.99
1.02
1.94
3.00
0.5 10.0 9.5 9.0 8.5 8.0 7.5 7.0 6.5 6.0 5.5 5.0 4.5 4.0 3.5 3.0 2.5 2.0 1.5 1.0 0.5 0.0
f1 (ppm)

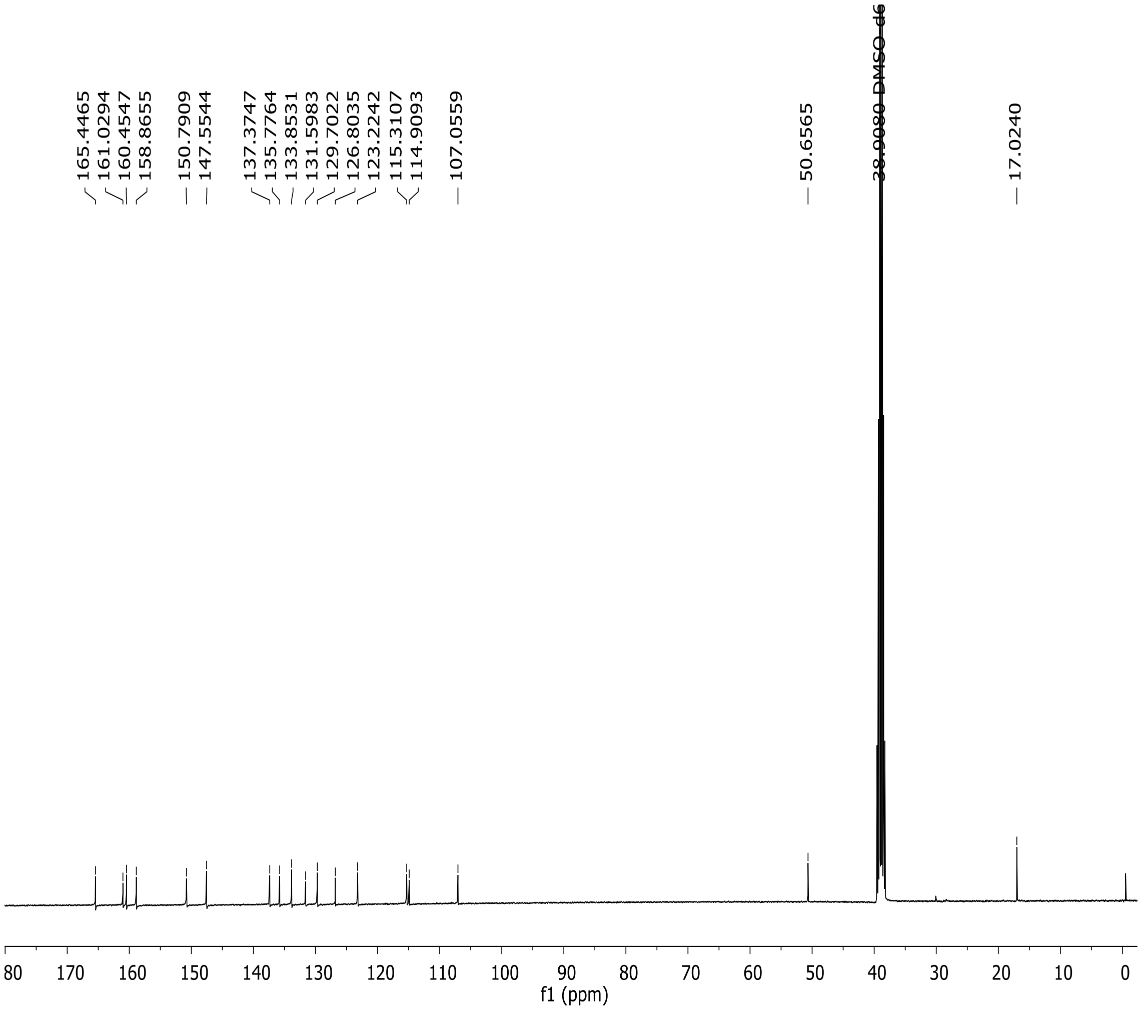

General procedure for the synthesis of compounds 1a, b, and 2a–j

To a flask, 1 mmol of the azide compound (0.303 g (**5**) or 0.360 g (**9**)) and 12 mL of an acetonitrile/water mixture (2:1) were added, followed by sodium ascorbate (0.5 mmol, 0.099 g), $CuSO_4 \cdot 5H_2O$ (0.1 mmol, 0.025 g, 10 mol %) and 1.5 mmol of the properly substituted acetylene **6a–j**, and the reaction mixture was irradiated in a microwave reactor [5]. The reaction progress was monitored by TLC using a chloroform/methanol 95:5 mixture as the eluent. The target compounds were purified by filtration, recrystallization, or column chromatography.

(1-(4-Methyl-3-((4-(pyridin-3-yl)pyrimidin-2-yl)amino)phenyl)-1*H*-1,2,3-triazol-4-yl)methanol (1a)

MW conditions: 8 min, 100 W, 80 °C. White solid; Yield: 84%, m.p.:194-196 °C (recrist. from acetonitrile). The analytical data corresponded to the literature [6,7].

3-(1-(4-Methyl-3-((4-(pyridin-3-yl)pyrimidin-2-yl)amino)phenyl)-1*H*-1,2,3-triazol-4-yl)propan-1-ol (1b)

MW conditions: 20 min, 100 W, 80 °C. White solid; Yield: 76%, m.p.: 64.7-65.6 °C (recrist. from acetonitrile). IR (cm^{-1}; film): 3401 (OH str.); 1039 (C-C-O str.). ^{1}H NMR (DMSO-d_6, 400 MHz): 1.77-1.87 (m, 2H, CH_2),2.35 (s, 3H, CH_3), 2.68-2.80 (m, 2H, CH_2), 3.49 (m, 2H, CH_2), 4.54 (t, 1H, *J*= 5.1 Hz, OH), 7.43 (d, 1H, *J*= 8.4 Hz, Ar-H), 7.51-7.57 (m, 3H, Ar-H, H-pyrimidine, H-pyridine), 8.33 (d, 1H, *J*= 2.1 Hz, Ar-H), 8.48-8.52 (m, 1H, H-pyridine), 8.53 (s, 1H, H-triazole), 8.58 (d, 1H, *J*= 5.2 Hz, H-pyrimidine), 8.71 (dd, 1H, *J*= 4.7, 1.4 Hz, H-pyridine), 9.14 (s, 1H, NH), 9,30 (d, 1H, *J*= 1.8 Hz, H-pyridine). ^{13}C NMR (DMSO-d_6, 101 MHz): 17.18, 21.10, 31.50, 59.42, 107.65, 114.37, 114.77, 119.83, 123.24, 130.70, 130.84, 131.47, 133.78, 134.31, 138.38, 147.38, 147.56, 150.93, 159.01, 160.16, 161.03. Anal. Calcd. (%) for CHN: C, 65.10; H, 5.46; N, 25.31; Found (%): C, 64.98; H, 5.47; N, 25.29. HR-MS (ESI) *m/z* calculated for $C_{21}H_{21}N_7ONa$: 410.1705 $[M+Na]^+$; found: 410.1694 $[M+Na]^+$.

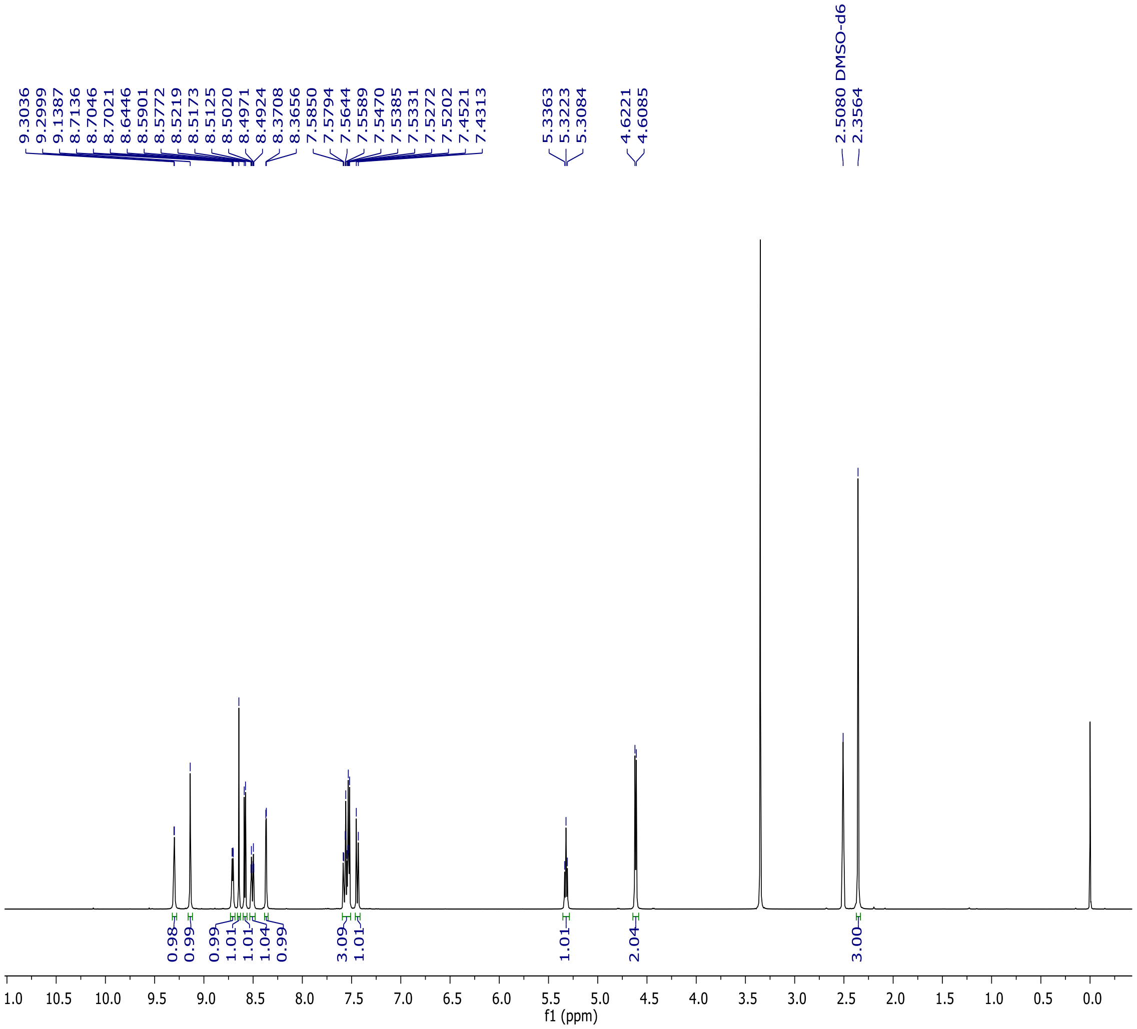

9.3036
9.2999
9.1387
8.7136
8.7046
8.7021
8.6446
8.5901
8.5772
8.5219
8.5173
8.5125
8.5020
8.4971
8.4924
8.3708
8.3656
7.5850
7.5794
7.5644
7.5589
7.5470
7.5385
7.5331
7.5272
7.5202
7.4521
7.4313
5.3363
5.3223
5.3084
4.6221
4.6085
2.5080 DMSO-d6
2.3564
0.98
0.99
0.99
1.01
1.01
1.04
0.99
3.09
1.01
1.01
2.04
3.00
f1 (ppm)

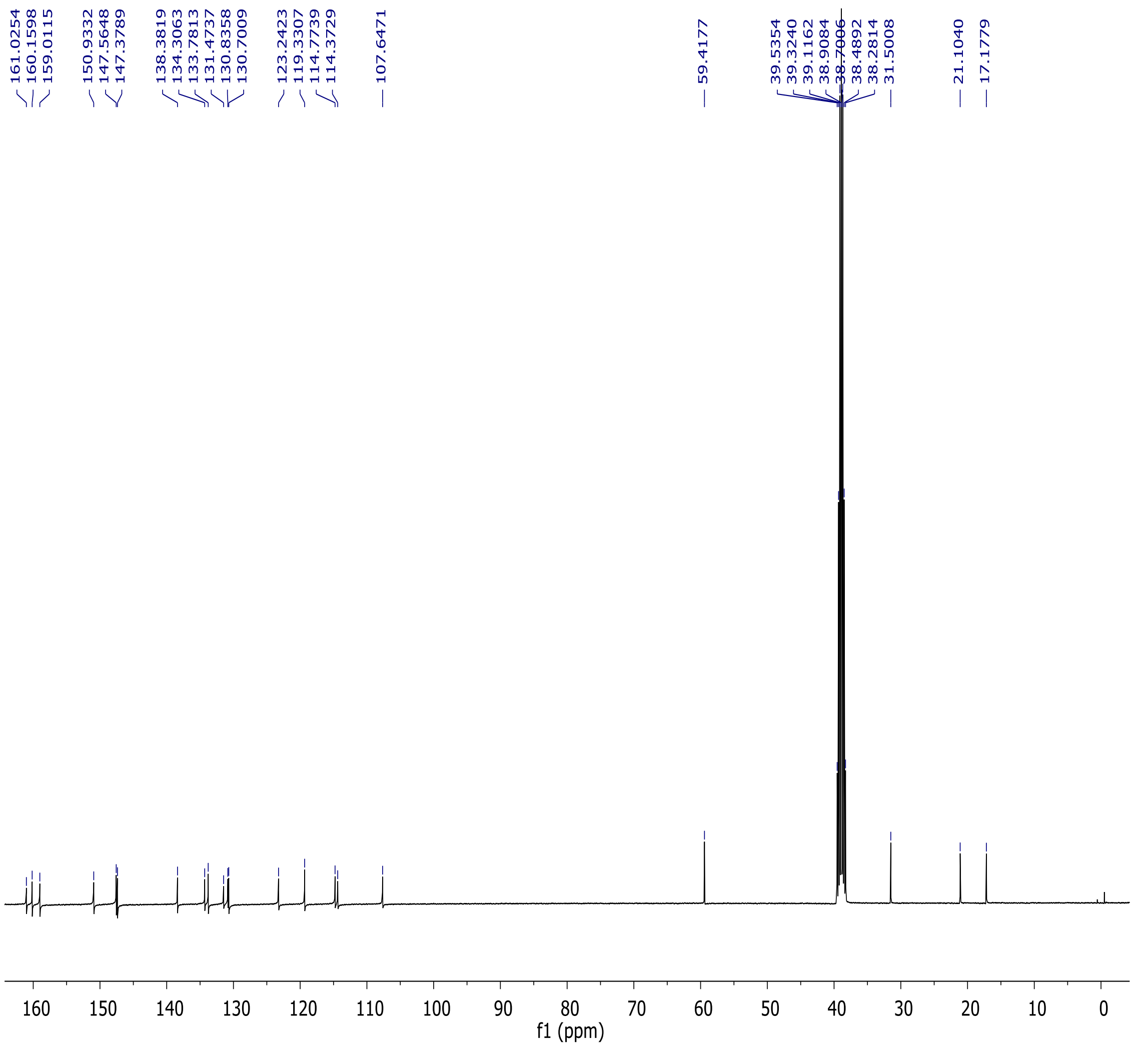

2-(4-(Hydroxymethyl)-1*H*-1,2,3-triazol-1-yl)-*N*-(4-methyl-3-((4-(pyridin-3-yl)pyrimidin-2-yl)amino)phenyl)acetamide (2a)

MW conditions: 60 min, 100 W, 80 °C. Yellow solid; Yield: 75%, m.p.: 215-217 °C (recryst. from acetonitrile). IR (cm^{-1}; film): 3372 and 3260 (N-H str.); 1667 (C=O str.); 1009 (C-C-O str.). ^{1}H NMR (DMSO-d_6, 400 MHz): 2.21 (s, 3H, CH_3), 4.54 (d, 2H, *J*= 5.6 Hz, CH_2OH), 5.22 (t, 1H, *J*= 5.7 Hz, OH), 5.30 (s, 2H, CH_2), 7.19 (d, 1H, *J*= 8.3 Hz, Ar-H), 7.29 (dd, 1H, *J*= 8.2, 2.0 Hz, Ar-H), 7.44 (d, 1H, *J*= 5.2 Hz, H-pyrimidine), 7.50 (dd, 1H, *J*= 8.0, 4.8 Hz, H-pyridine), 7.94 (d, 1H, *J*= 1.7 Hz, Ar-H), 7.99 (s, 1H, H-triazole), 8.46 (dt, 1H, *J*= 8.0, 1.9 Hz, H-pyridine), 8.51 (d, 1H, *J*= 5.1 Hz, H-pyrimidine), 8.69 (d, 1H, *J* = 4.1 Hz, H-pyridine), 8.92

(s, 1H, NH), 9.25 (s, 1H, H-pyridine), 10.41 (s, 1H, NH). ^{13}C NMR (DMSO-d_6, 101 MHz): 18.09, 52.61, 55.49, 108.17, 115.80, 116.14, 124.34, 124.84, 127.73, 130.84, 132.64, 134.97, 136.87, 138.49, 148.25, 148.60, 151.87, 159.94, 161.47, 162.10, 164.54. Anal. Calcd. (%) for CHN: C, 60.57; H, 4.84; N, 26.91; Found (%): C, 60.45; H, 4.84; N, 26.88. HR-MS (ESI) *m/z* calculated for $C_{21}H_{20}N_8O_2Na$: 439.1607 $[M+Na]^+$; found: 439.1588 $[M+Na]^+$.

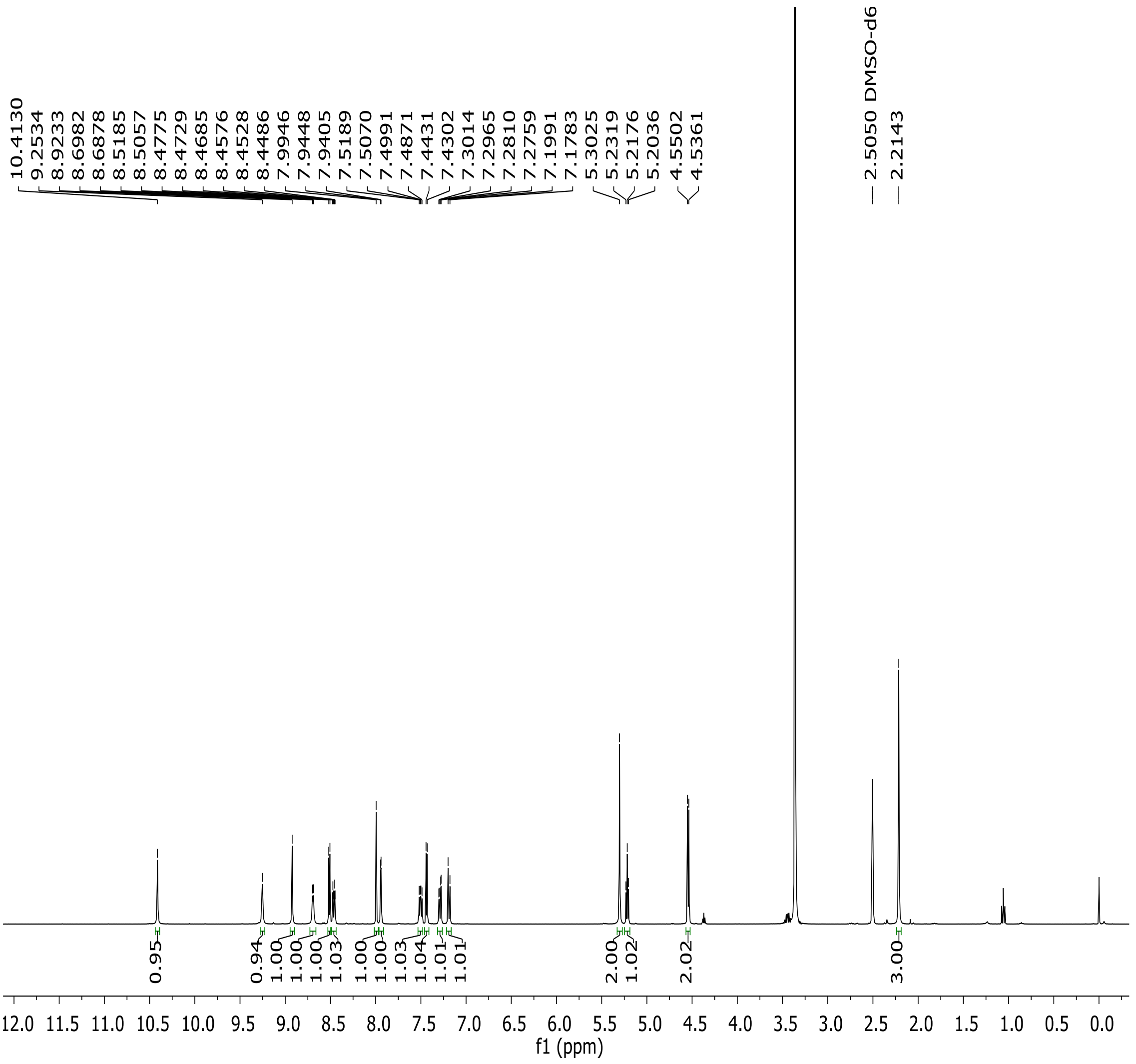

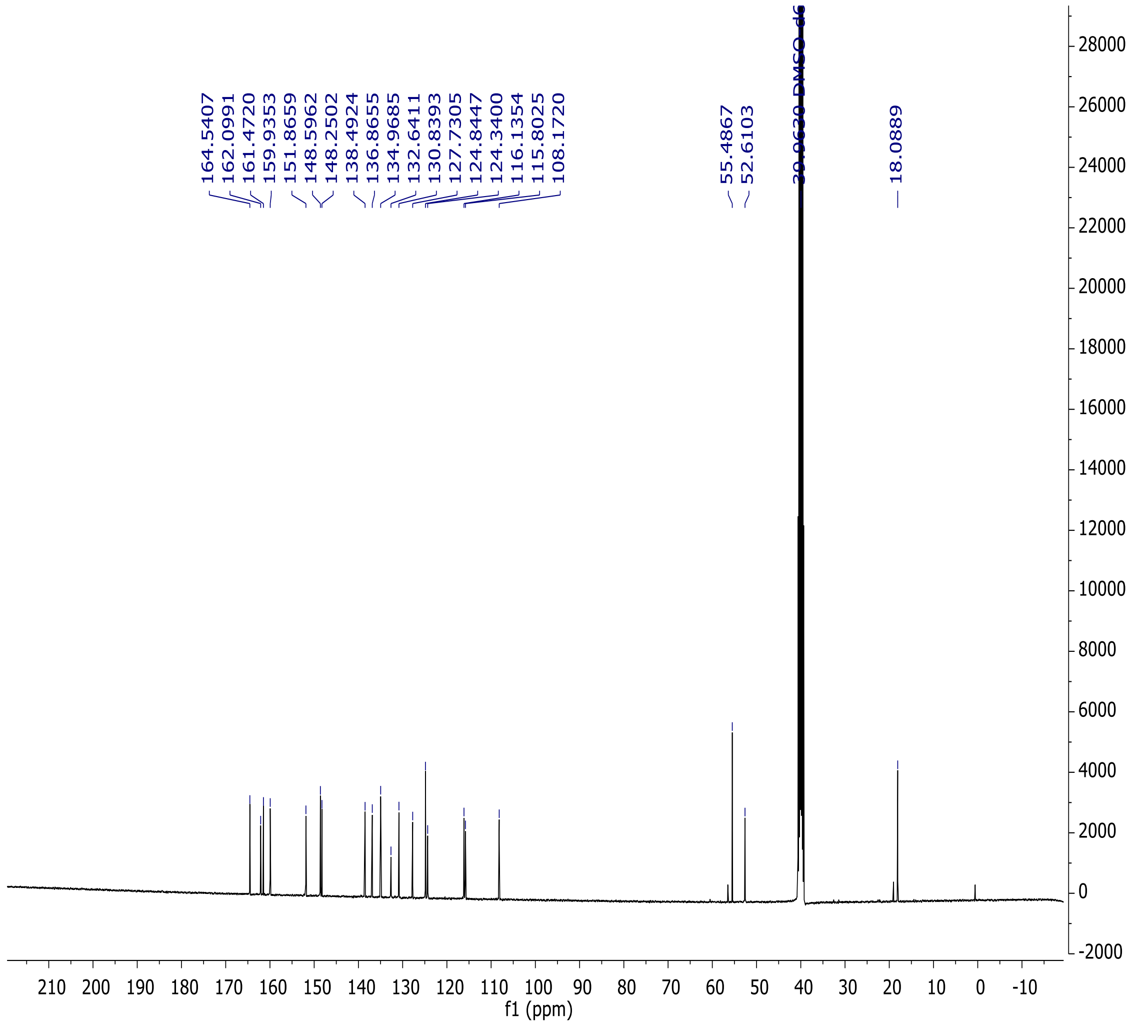

2-(4-(3-Hydroxypropyl)-1*H*-1,2,3-triazol-1-yl)-*N*-(4-methyl-3-((4-(pyridin-3-yl)pyrimidin-2-yl)amino)phenyl)acetamide (2b)

MW conditions: 80 min, 100 W, 80 °C. Yellow solid; Yield: 80%, m.p.: 170-172 °C (recryst. from acetonitrile). IR (cm^{-1}; film): 3270 (N-H str.); 1670 (C=O str.); 1009 (C-C-O str.).^{1}H NMR (DMSO-d_6, 400 MHz): 2.22 (s, 3H, CH_3), 2.67 (t, 2H, *J*= 7.6 Hz, CH_2), 3.45 (q, 2H, *J*= 6.2 Hz, CH_2), 4.50 (t, 1H, *J*= 5.1 Hz, OH), 5.27 (s, 2H, CH_2), 7.19 (d, 1H, *J*= 8.3 Hz, Ar-H), 7.28 (dd, 1H, *J*= 8.2, 1.9 Hz, Ar-H), 7.43 (d, 1H, *J*= 4.9 Hz, H-pyrimidine), 7.88 (s, 1H, H-triazole), 7.96 (m, 1H, Ar-H), 8.51 (d, 2H, *J* = 7.9 Hz, H-pyridine), 8.92 (s, 1H, NH), 10.40 (1H, s, NH). ^{13}C NMR (DMSO-d_6, 101 MHz): 17.01, 21.04, 31.69, 51.51, 59.44, 107.17, 114.69, 115.02,

122.95, 126.59, 129.74, 133.58, 135.80, 137.41, 146.16, 147.67, 150.67, 158.86, 160.41, 161.21, 163.52. Anal. Calcd. (%) for CHN: C, 62.15; H, 5.44; N, 25.21; Found (%): C, 62.08; H, 5.45; N, 25.36. HR-MS (ESI) *m/z* calculated for $C_{23}H_{24}N_8O_2Na$: 467.1920 [M+Na]$^+$; found: 467.1907 [M+Na]$^+$.

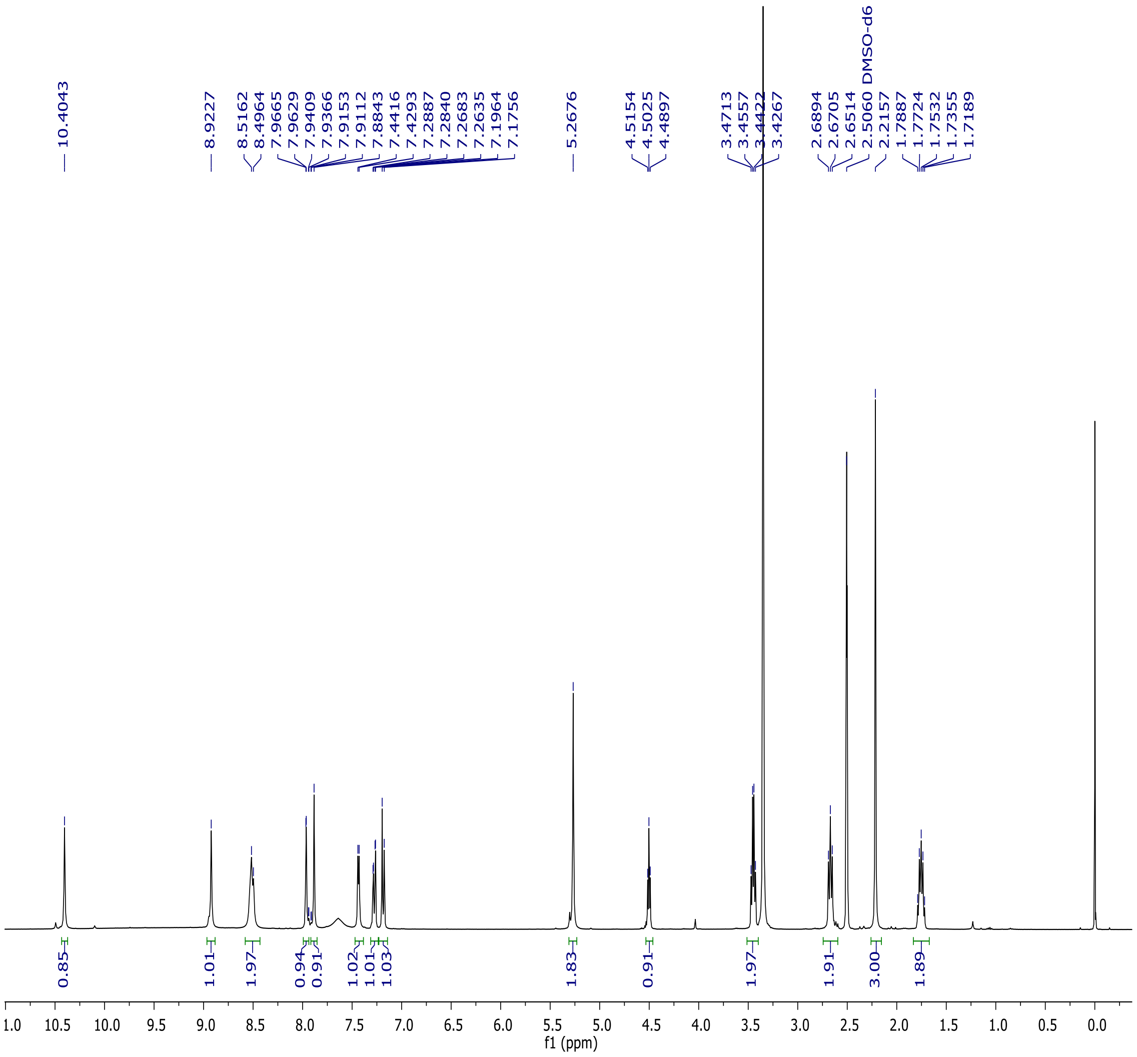

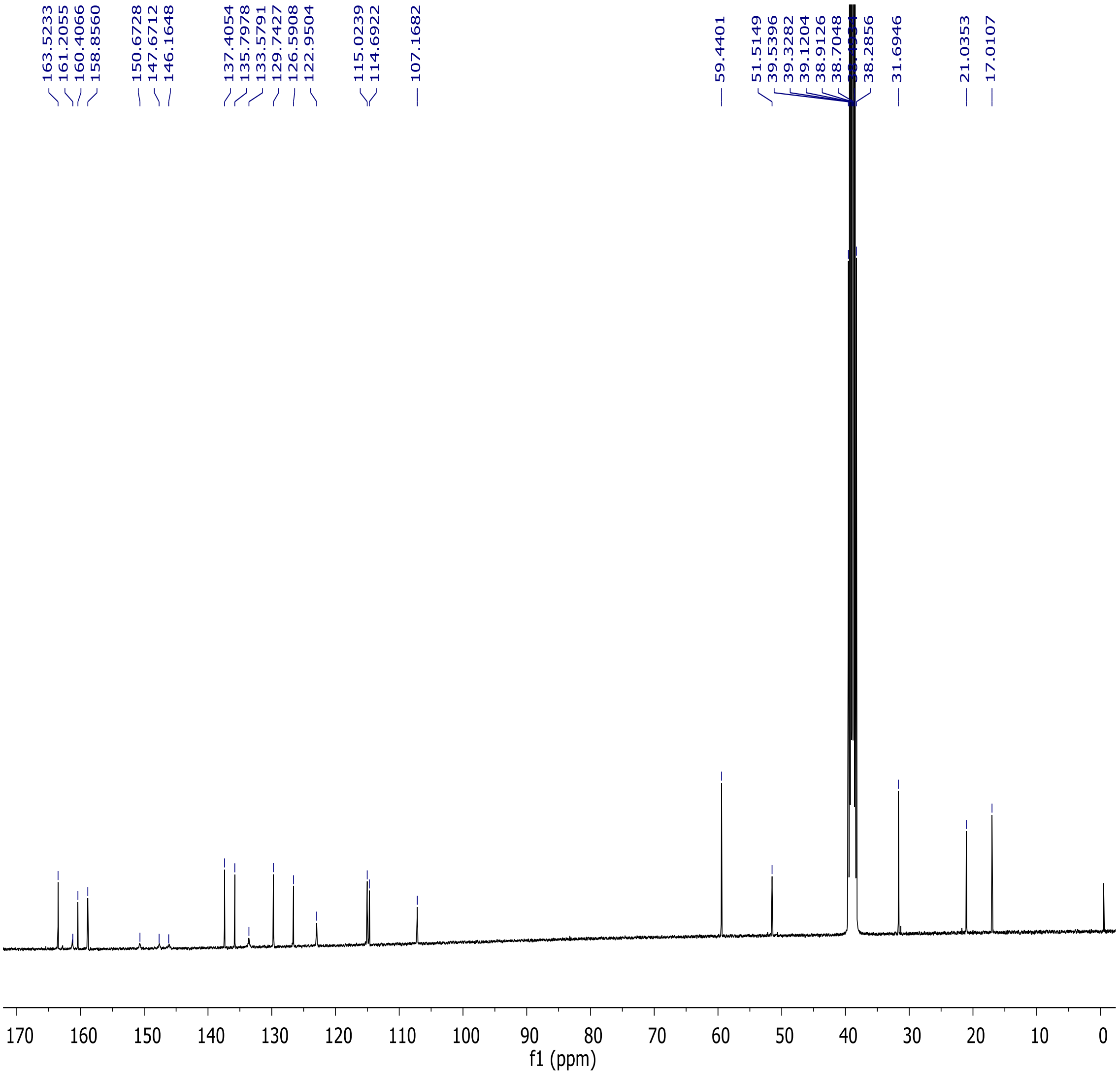

2-(4-Butyl-1*H*-1,2,3-triazol-1-yl)-*N*-(4-methyl-3-((4-(pyridin-3-yl)pyrimidin-2-yl)amino)phenyl)acetamide (2c)

MW conditions: 1 min, 100 W, 80 °C. Yellow solid; Yield: 80%, m.p.: 153-154 °C. IR (cm^{-1}; film): 3384 and 3263 (N-H str.); 1670 (C=O str). ^{1}H NMR (DMSO-d_6, 400 MHz): 0.89 (t, 3H, *J*= 7.4 Hz, CH_3), 1.33 (m, 2H, CH_2), 1.58 (p, 2H, *J* = 7.5 Hz, CH_2), 2.21 (s, 3H, CH_3), 2.63 (t, 2H, *J* = 7.6 Hz, CH_2), 5.26 (s, 2H, CH_2), 7.18 (d, 1H, *J* = 8.4 Hz,Ar-H), 7.27 (dd, 1H, *J* = 8.2, 2.1 Hz, Ar-H), 7.44 (d, 1H, *J* = 5.2 Hz, H-pyrimidine), 7.49 (dd, 1H, *J*= 7.9, 4.8 Hz, H-pyridine), 7.87 (s, 1H, H-triazole), 7.96 (d, 1H, *J*= 1.9 Hz, Ar-H), 8.46 (dt, 1H, *J*= 8.0, 1.8 Hz, H-pyridine), 8.51 (d, 1H,*J*= 5.1 Hz, H-pyrimidine), 8.69 (s, 1H, H-pyridine), 8.92 (s, 1H, NH),9.26 (s,

1H, H-pyridine), 10.40 (s, 1H, NH). ^{13}C NMR (DMSO-d_6, 101 MHz): 14.17, 17.50, 22.12, 25.10, 31.60, 52.58, 108.18, 115.52, 116.11, 123.92, 124.33, 127.67, 130.82, 132.77, 134.98, 136.88, 138.49, 147.12, 148.64, 151.83, 159.96, 161.47, 162.09, 164.61. HR-MS(ESI) *m/z* calculated for $C_{24}H_{26}N_8ONa$: 465.2127 $[M+Na]^+$; found: 465.2121 $[M+Na]^+$. HPLC-UV % (nm)***:*** 99.5 (264).

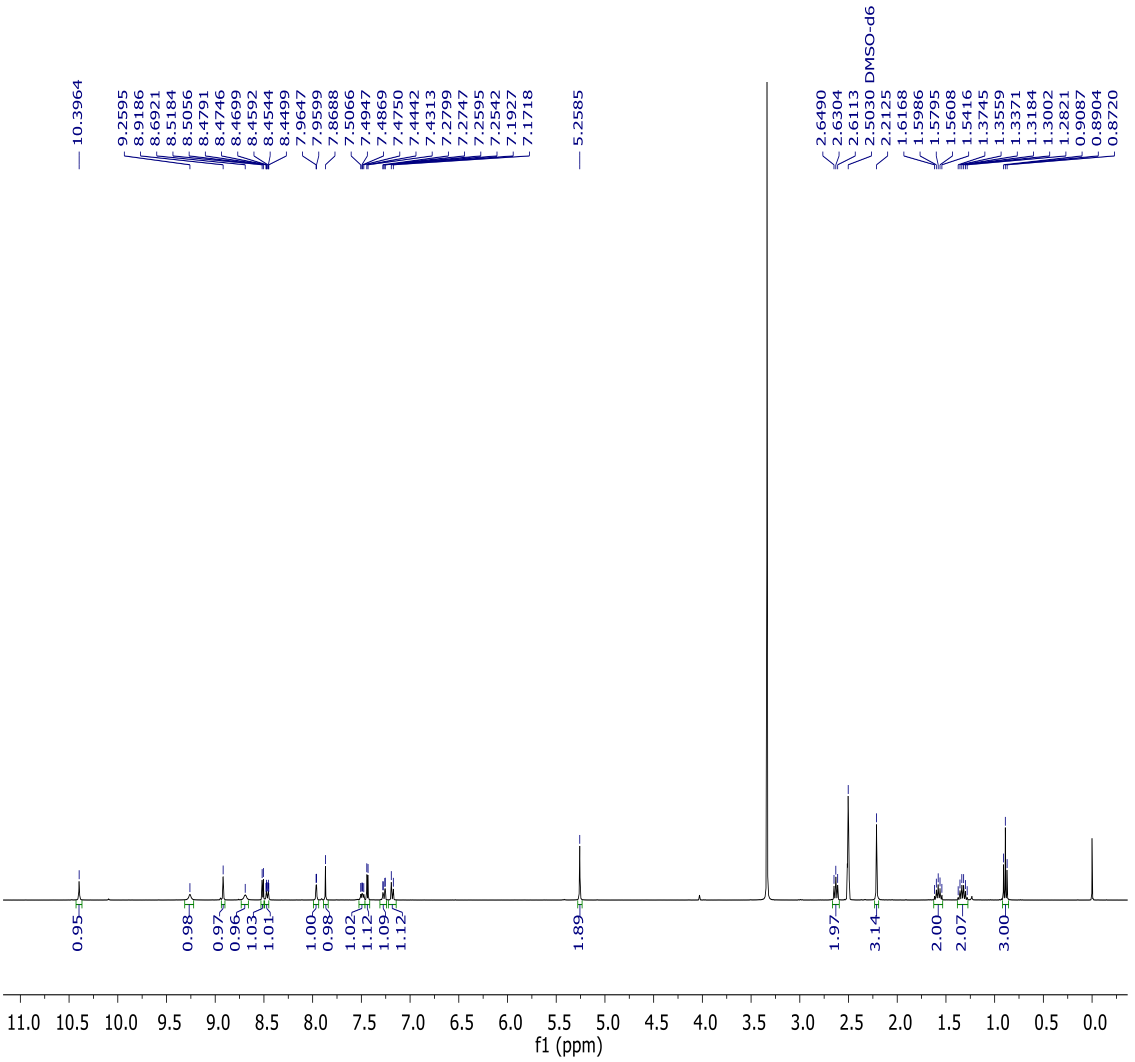

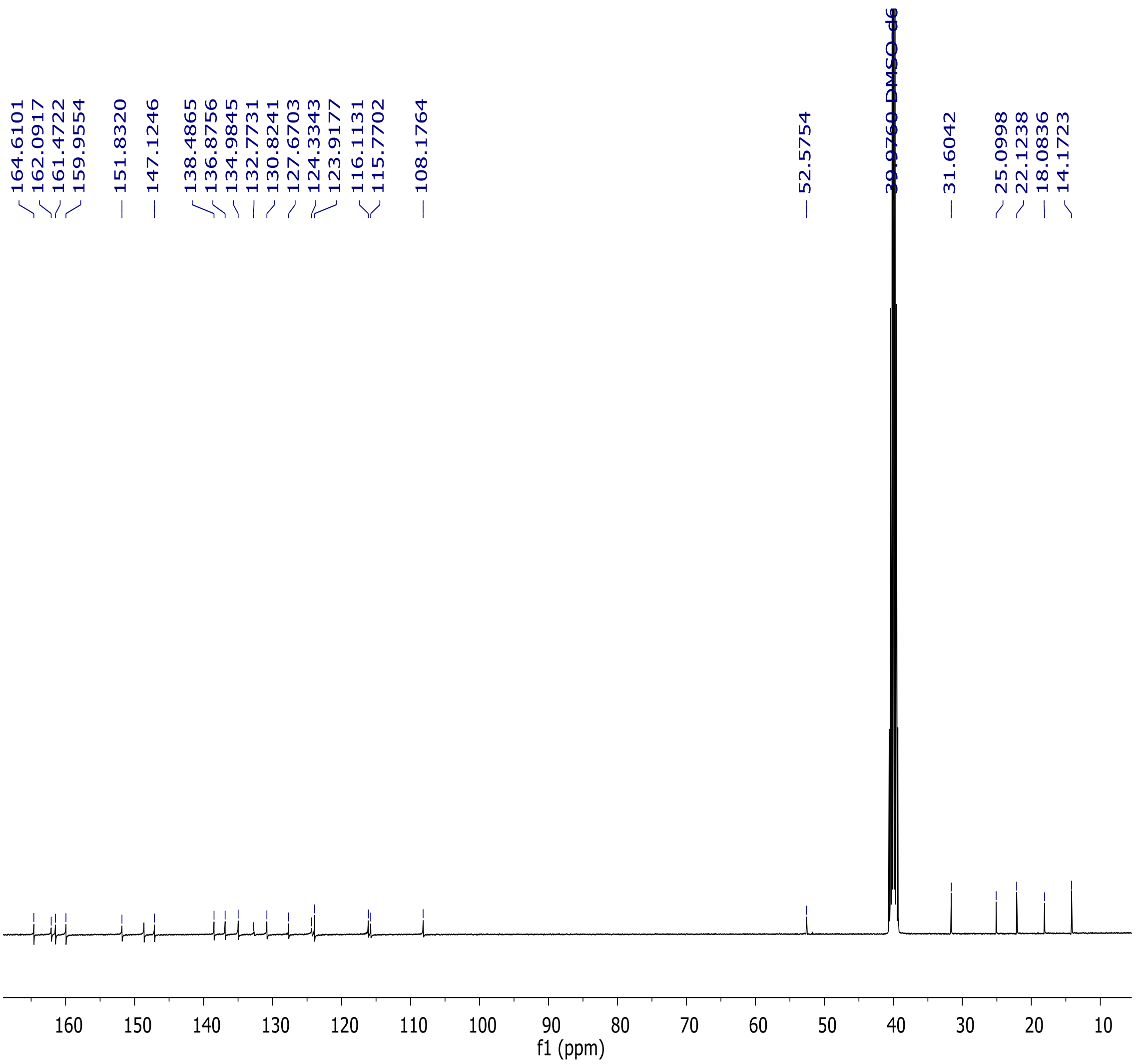

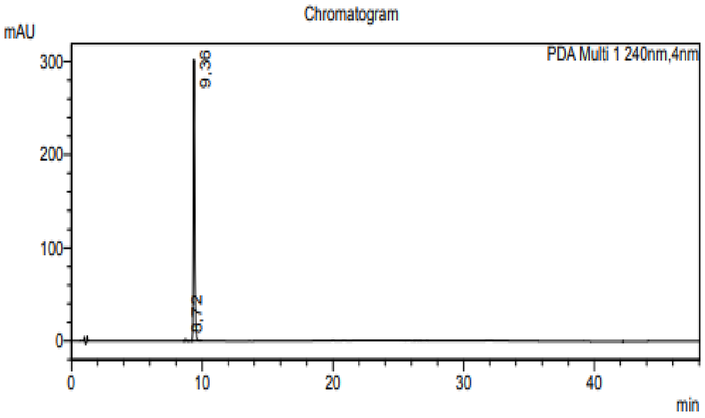

PDA Ch2 264nm

Peak#	Ret. Time	Name	Area	Area%	Theoretical Plates/meter(USP)	Tailing Factor	Resolution(USP)	Capacity Factor(k')
1	8,72		9000	0,5	182234	1,262	--	--
2	9,36		1755151	99,5	226250	1,400	3,112	0,074
Total			1764151	100,0				

2-(4-(3-Chloropropyl)-1*H*-1,2,3-triazol-1-yl)-*N*-(4-methyl-3-((4-(pyridin-3-yl)pyrimidin-2-yl)amino)phenyl)acetamide (2d)

MW conditions: 1 min, 100 W, 80 °C. Yellow solid; yield: 80%, m.p.: 134-136 °C (filtration). IR (cm^{-1}; film): 3264 (N-H str.); 1669 (C=O str.); 687 (C-Clstr.). ^{1}H NMR (DMSO-d_6, 400 MHz): 1.98-2.11 (m, 2H, CH_2), 2.22 (s, 3H, CH_3), 2.79 (t, 2H, J = 7.4 Hz, CH_2), 3.69 (t, 2H, J = 6.4 Hz, CH_2), 5.29 (s, 2H, CH_2), 7.19 (d, 1H, J = 8.3 Hz,Ar-H), 7.28 (d, 1H, J = 7.8 Hz, Ar-H), 7.43 (d, 1H, J = 4.3 Hz, H-pyrimidine), 7.69 (s, 1H, H-pyridine), 7.96 (d, 2H, J = 8.3 Hz, Ar-H, H-triazole), 8.53 (s, 2H, H-pyrimidine, H-pyridine), 8.92 (s, 1H, NH), 10.42 (s, 1H, NH). ^{13}C NMR (DMSO-d_6, 101 MHz): 17.01, 21.66, 31.20, 44.08, 51.54, 107.27, 114.69, 115.02, 123.25, 126.58, 129.74, 133.32, 135.78, 137.40, 144.73, 147.71, 150.51, 158.83, 160.43, 161.47, 163.48. HR-MS (ESI) *m/z* calculated for $C_{23}H_{23}ClN_8ONa$: 485.1581 $[M+Na]^+$; found: 485.1574 $[M+Na]^+$. HPLC-UV % (nm)***:*** 97.1 (264).

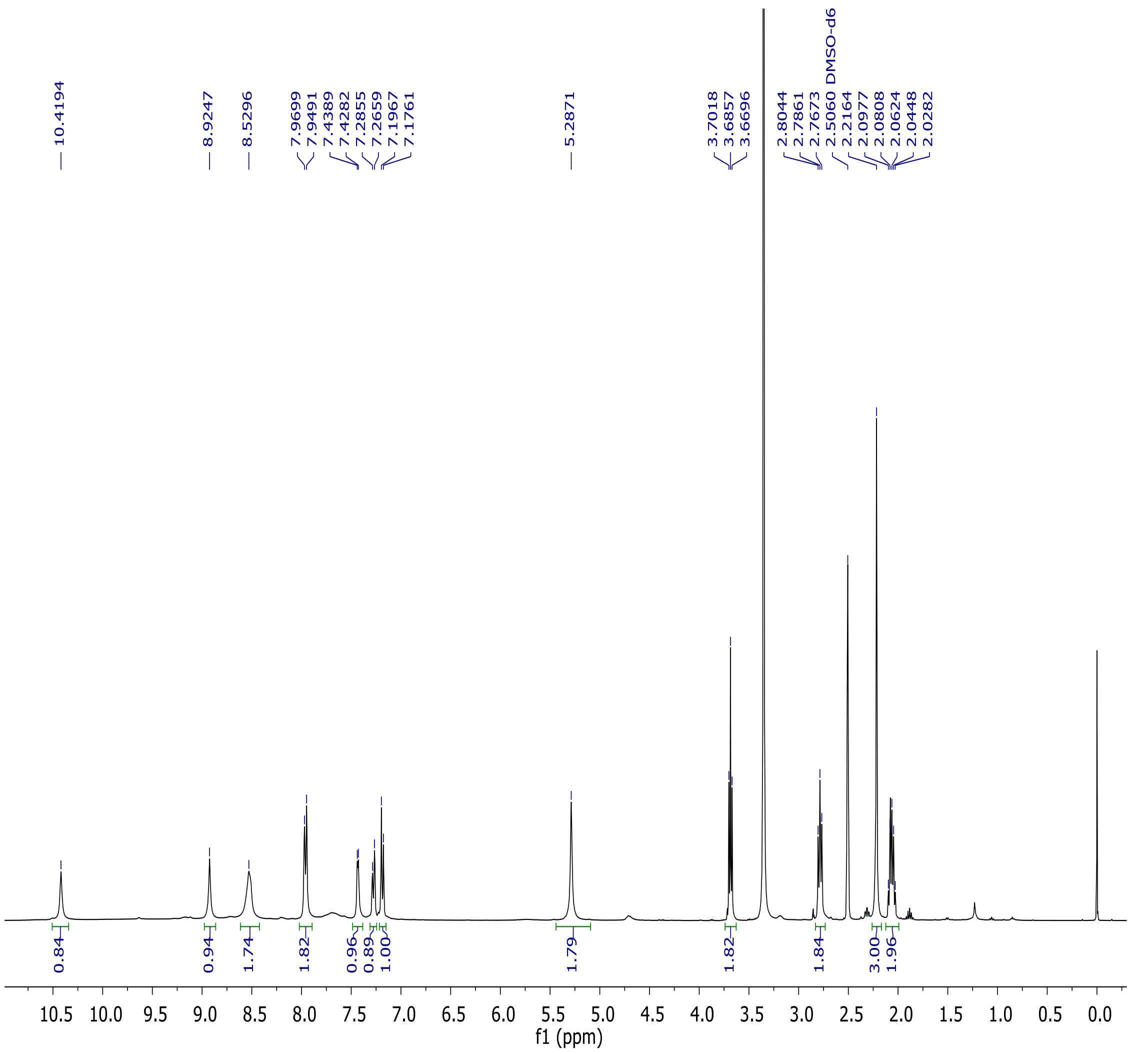
10.4194
8.9247
8.5296
7.9699
7.9491
7.4389
7.4282
7.2855
7.2659
7.1967
7.1761
5.2871
3.7018
3.6857
3.6696
2.8044
2.7861
2.7673
2.5060 DMSO-d6
2.2164
2.0977
2.0808
2.0624
2.0448
2.0282
0.84
0.94
1.74
1.82
0.96
0.89
1.00
1.79
1.82
1.84
3.00
1.96
f1 (ppm)

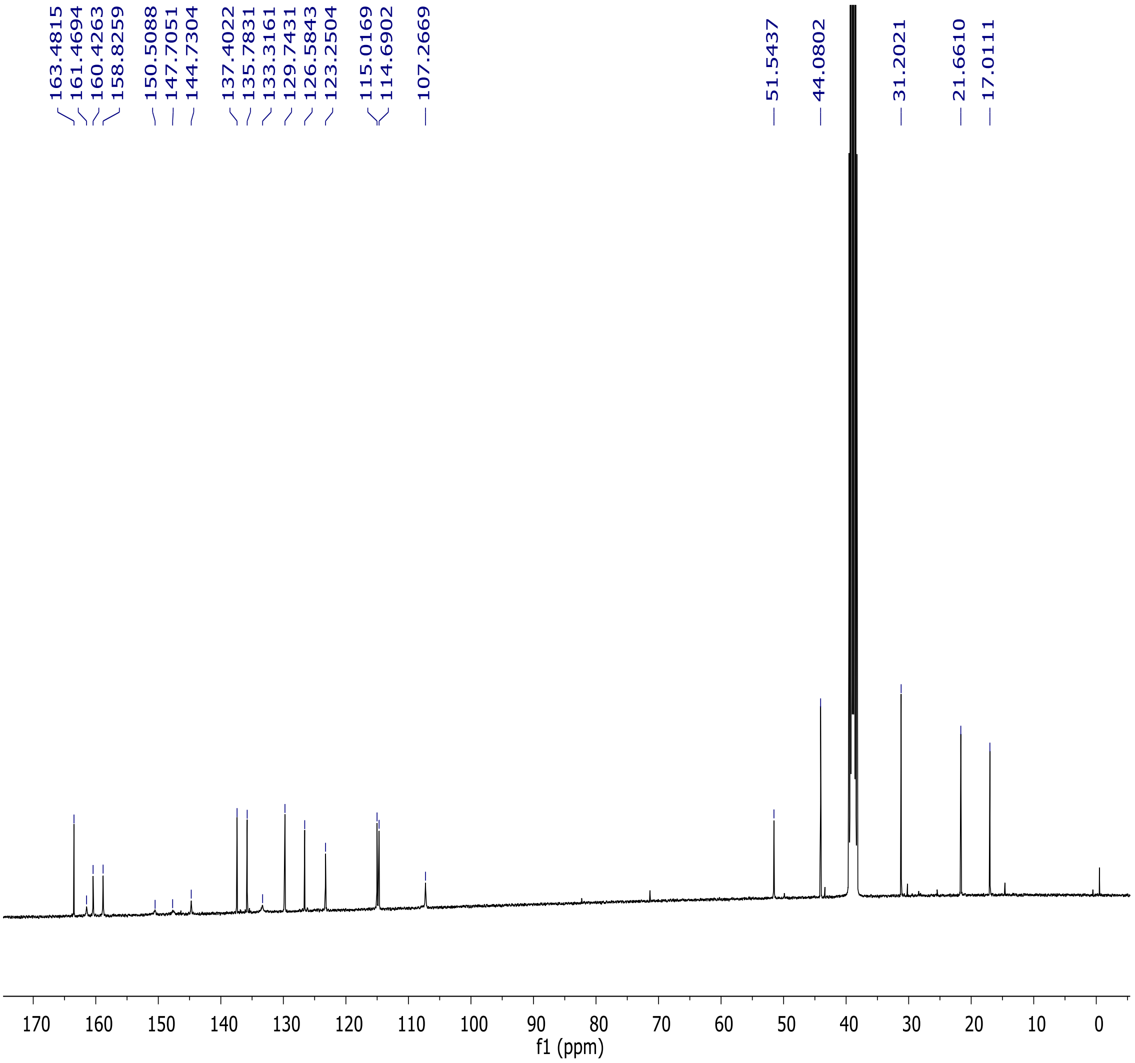
163.4815
161.4694
160.4263
158.8259
150.5088
147.7051
144.7304
137.4022
135.7831
133.3161
129.7431
126.5843
123.2504
115.0169
114.6902
107.2669
51.5437
44.0802
31.2021
21.6610
17.0111
170
160
150
140
130
120
110
100
90
80
70
60
50
40
30
20
10
0
f1 (ppm)

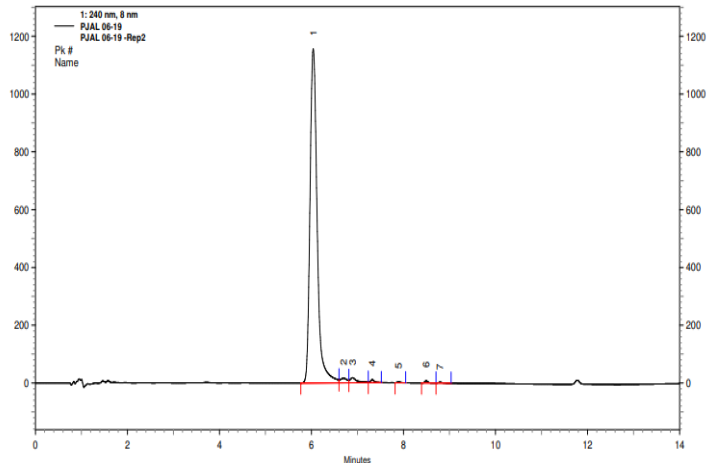
1: 240 nm, 8 nm
PJAL 06-19
PJAL 06-19 -Rep2
Pk #
Name
1
2
3
4
5
6
7
1200
1000
800
600
400
200
0
0
2
4
6
8
10
12
14
Minutes

2: 264 nm, 8 nm

Pk #	Retention Time	Area	Area %	Height	Height %	Width
1	6.040	10654940	97.108	1000546	95.727	0.80
2	6.696	112364	1.024	11660	1.116	0.21
3	6.896	108220	0.986	13153	1.258	0.35
4	7.320	38546	0.351	7627	0.730	0.26
5	7.900	9662	0.088	2101	0.201	0.20
6	8.496	35080	0.320	7582	0.725	0.23
7	8.796	13476	0.123	2542	0.243	0.26
Totals		10972288	100.000	1045211	100.000	

2-(4-Cyclopropyl-1*H*-1,2,3-triazol-1-yl)-*N*-(4-methyl-3-((4-(pyridin-3-yl)pyrimidin-2-yl)amino)phenyl)acetamide (2e)

MW conditions: 30 min, 65 W, 70 °C (sealed tube). White solid; yield: 30%, m.p.: 165-167 °C (99:1 DCM/MeOH). IR (cm^{-1}; film): 3364 (N-H str.); 1660 (C=O str.). ^{1}H NMR (MeOD, 400 MHz): 0.73-0.84 (m, 2H, CH_2), 0.92-1.07 (m, 2H, CH_2), 1.99 (tt, 1H, *J* = 8.5, 5.0 Hz, CH), 2.29 (s, 3H, CH_3), 5.28 (s, 2H, CH_2), 7.17-7.25 (m, 2H, Ar-H), 7.36 (d, 1H, *J* = 5.2 Hz, H-pyrimidine), 7.54 (s, 1H, H-pyridine), 7.79 (s, 1H, H-triazole), 8.23 (d, 1H, *J* = 1.3 Hz, Ar-H), 8.46 (d, 1H, *J* = 4.8 Hz, H-pyrimidine), 8.60 (d, 1H, *J* = 7.9 Hz, H-pyridine). ^{13}C NMR (MeOD, 101 MHz): 7.28, 8.24, 17.89, 53.63, 108.92, 116.70, 117.19, 123.92, 125.65, 128.50, 131.74, 137.04, 137.60, 139.23, 148.99, 151.48, 151.72, 160.52, 162.60, 163.78, 165.84. HR-MS (ESI) *m/z* calculated for $C_{23}H_{22}N_8ONa$: 449.1814 $[M+Na]^+$; found: 449.1792 $[M+Na]^+$. HPLC-UV % (nm): 97.5 (264).

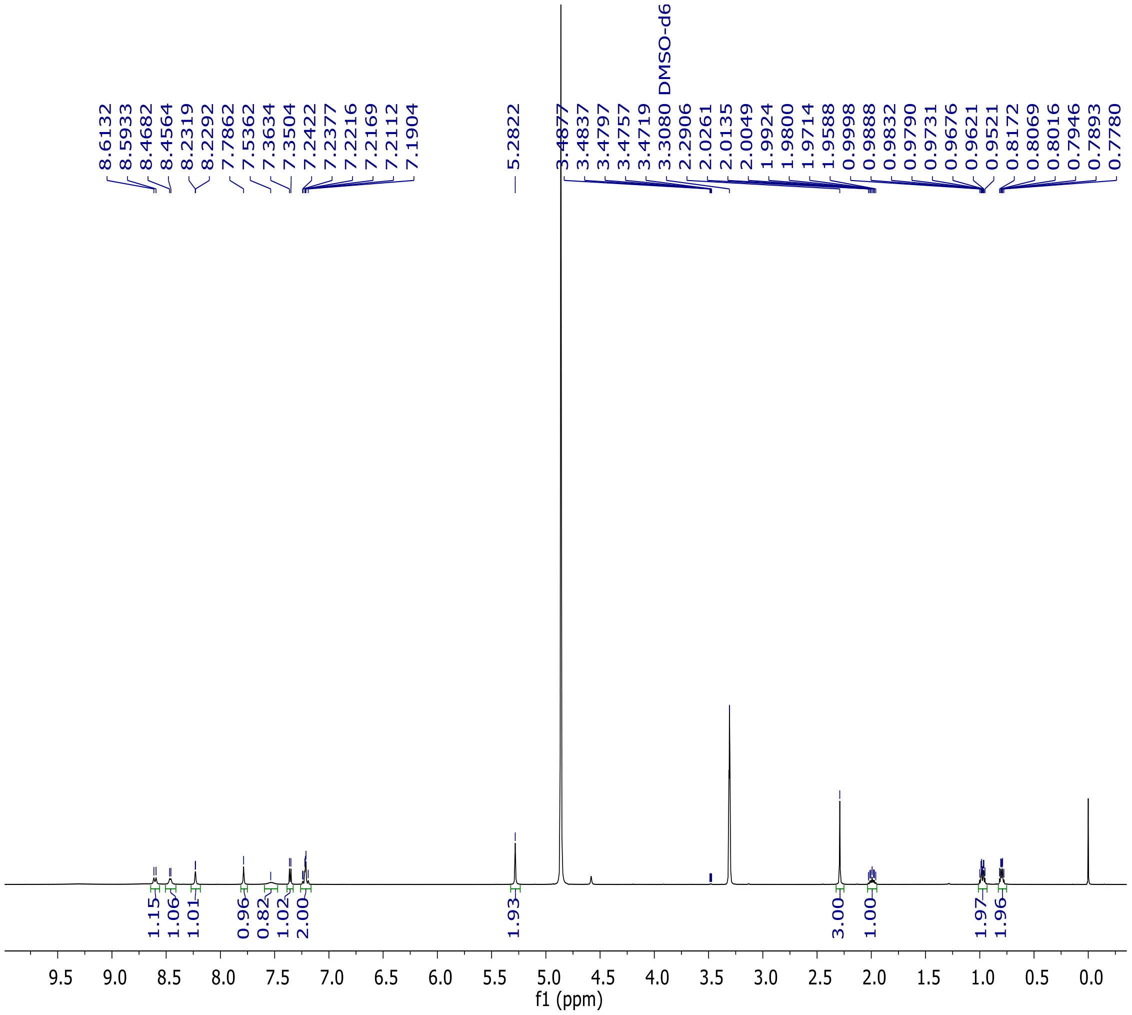
8.6132
8.5933
8.4682
8.4564
8.2319
8.2292
7.7862
7.5362
7.3634
7.3504
7.2422
7.2377
7.2216
7.2169
7.2112
7.1904
5.2822
3.4877
3.4837
3.4797
3.4757
3.4719
3.3080 DMSO-d6
2.2906
2.0261
2.0135
2.0049
1.9924
1.9800
1.9714
1.9588
0.9998
0.9888
0.9832
0.9790
0.9731
0.9676
0.9621
0.9521
0.8172
0.8069
0.8016
0.7946
0.7893
0.7780
1.15
1.06
1.01
0.96
0.82
1.02
2.00
1.93
3.00
1.00
1.97
1.96
9.5 9.0 8.5 8.0 7.5 7.0 6.5 6.0 5.5 5.0 4.5 4.0 3.5 3.0 2.5 2.0 1.5 1.0 0.5 0.0
f1 (ppm)

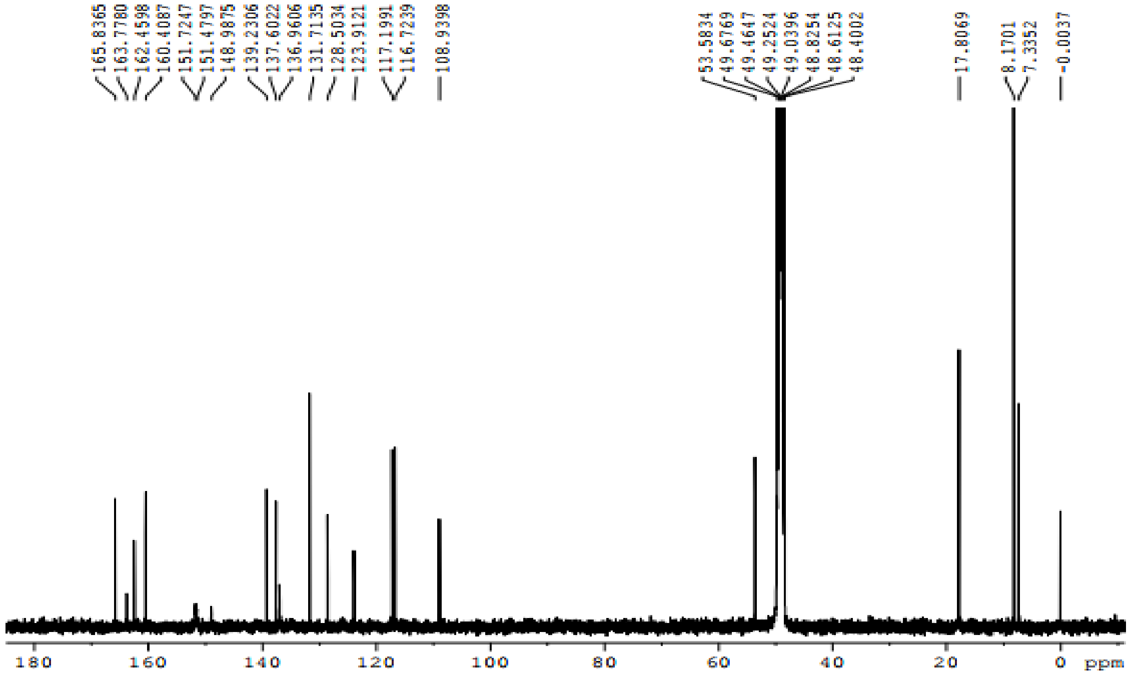
165.8365
163.7780
162.4598
160.4087
151.7247
151.4797
148.9875
139.2306
137.6022
136.9606
131.7135
128.5034
123.9121
117.1991
116.7239
108.9398
53.5834
49.6769
49.4647
49.2524
49.0396
48.8254
48.6125
48.4002
17.8069
8.1701
7.3352
-0.0037
180 160 140 120 100 80 60 40 20 0 ppm

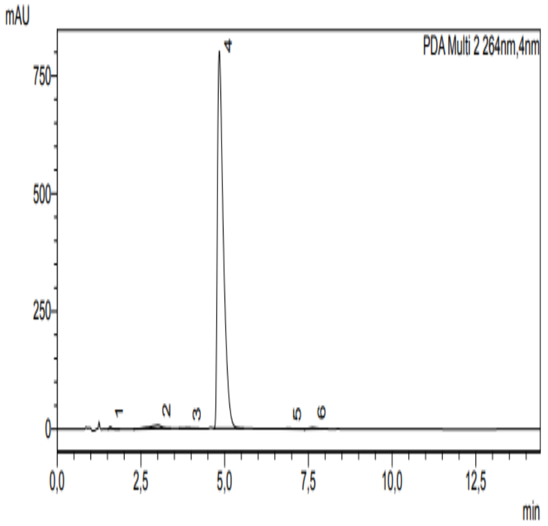

PDA Ch2 264nm

Peak#	Ret. Time	Name	Area	Area%	Theoretical Plates/meter(USP)	Tailing Factor	Resolution(USP)	Capacity Factor(k')
1	1,58		19797	0,2	12487	1,360	--	--
2	3,00		171614	1,7	2496	0,744	3,701	0,896
3	3,90		15603	0,2	7512	1,258	1,657	1,465
4	4,84		9956697	97,5	23183	1,971	2,367	2,057
5	6,91		15928	0,2	29974	1,587	5,576	3,361
6	7,63		32440	0,3	41151	1,302	1,811	3,819
Total			10212078	100,0				

2-(4-(1-Hydroxycyclohexyl)-1*H*-1,2,3-triazol-1-yl)-*N*-(4-methyl-3-((4-(pyridin-3-yl)pyrimidin-2-yl)amino)phenyl)acetamide (2f)

MW conditions: 40 min., 100 W, 80 °C (sealed tube). White solid; yield: 50%, m.p.: 220-222 °C (99:1 DCM/MeOH). IR (cm^{-1}; film): 3273 (N-H str.); 1681 (C=O str.). ^{1}H NMR (MeOD, 400 MHz): 1.33-1.45 (m, 1H, CH_2), 1.52 (dq, 2H, *J* = 13.9, 4.8 Hz, CH_2), 1.61 (dq, 1H, dq, *J* = 11.9, 4.8, 4.1 Hz, CH_2), 1.71-1.89 (m, 2H, CH_2), 1.81-1.88 (m, 2H, CH_2), 2.03 (td, 2H, *J* = 12.4, 10.8 Hz, 3.8 Hz, CH_2), 2.29 (s, 3H, CH_3), 5.32 (s, 2H, CH_2), 7.22 (d, 2H, *J* = 1.5 Hz, Ar-H), 7.36 (d, 1H, *J* = 5.2 Hz, H-pyrimidine), 7.47-7.57 (m, 1H, H-pyridine), 7.94 (s, 1H, H-triazole); 8.23 (s, 1H, Ar-H), 8.46 (d, 1H, *J* = 5.2 Hz, H-pyrimidine), 8.59 (dt, 1H,*J* = 8.1, 1.9 Hz, H-pyridine), 8.63 (dd, 1H, *J* = 4.8, 1.4 Hz, H-pyridine), 9.25 (d, 1H, *J*= 1.7 Hz, H-

pyridine). ^{13}C NMR (MeOD, 101 MHz): 17.81, 23.11, 26.64, 38.91, 53.61, 70.32, 108.92, 116.77, 117.21, 124.31, 125.57, 128.55, 131.74, 134.57, 137.03, 137.63, 139.25, 149.03, 151.52, 157.02, 160.04, 162.48, 163.74, 165.85. HR-MS (ESI) *m/z* calculated for $C_{26}H_{28}N_8O_2Na$: 507.2233 $[M+Na]^+$; found: 507.2216 $[M+Na]^+$. HPLC-UV % (nm): 100.0 (264).

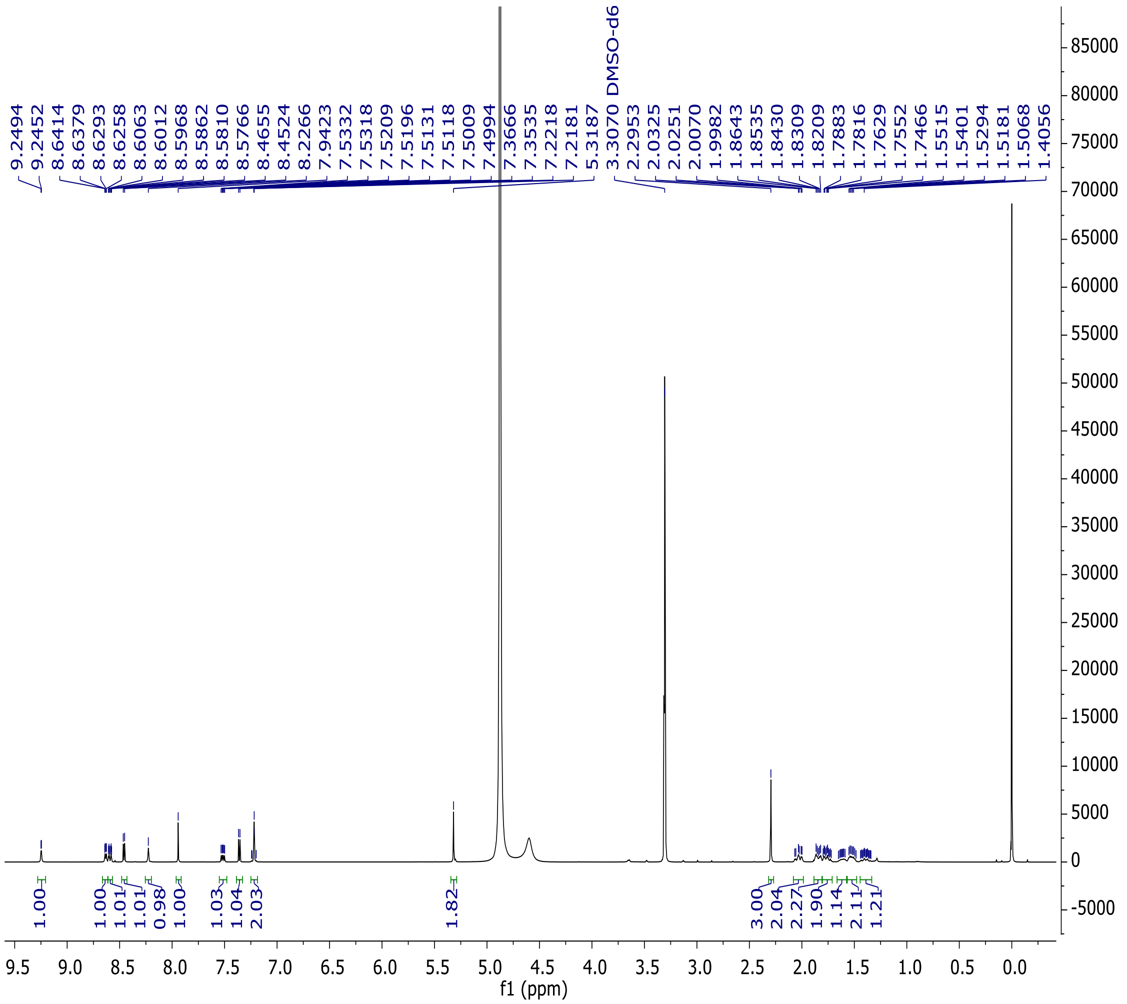

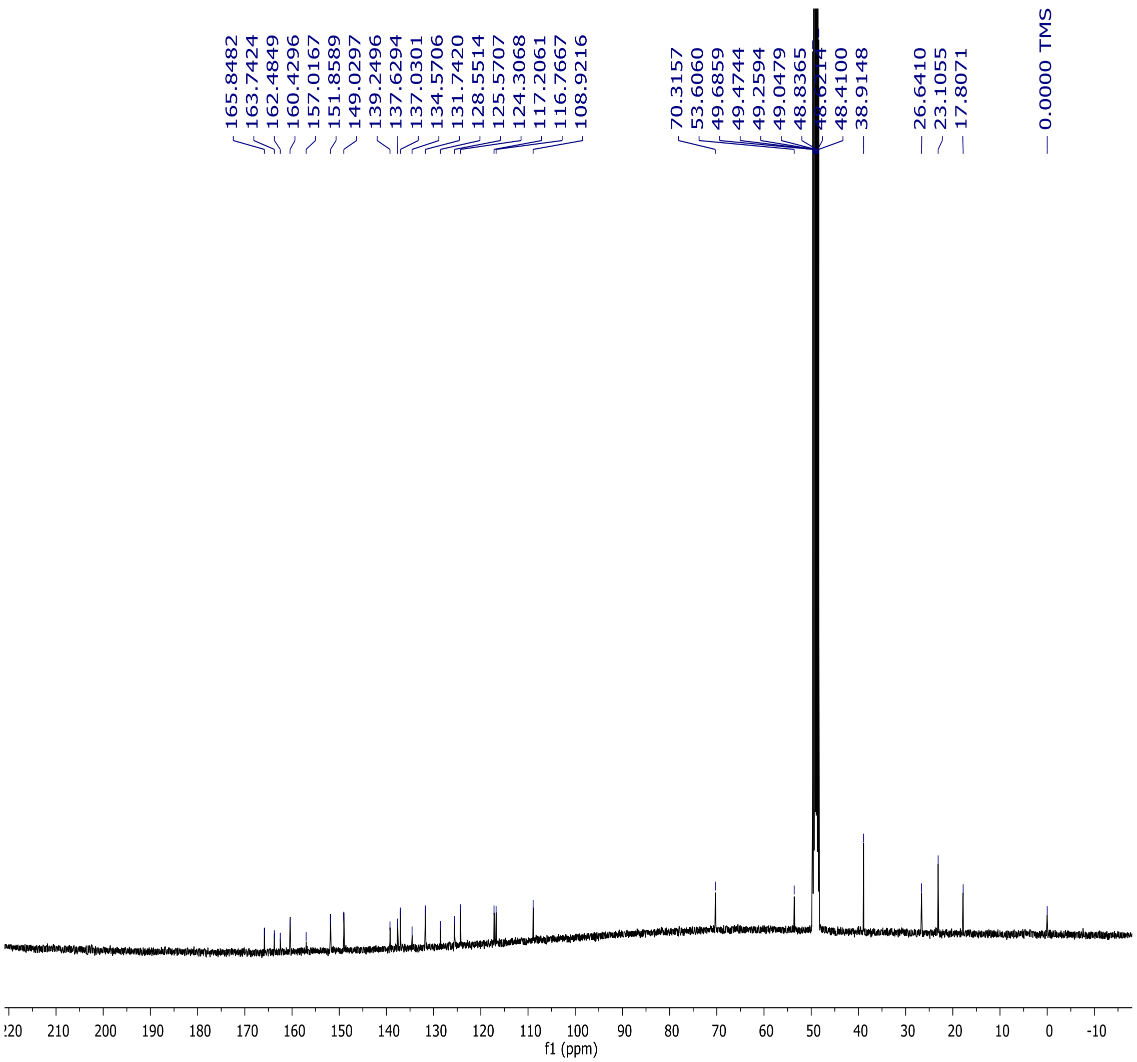
165.8482
163.7424
162.4849
160.4296
157.0167
151.8589
149.0297
139.2496
137.6294
137.0301
134.5706
131.7420
128.5514
125.5707
124.3068
117.2061
116.7667
108.9216
70.3157
53.6060
49.6859
49.4744
49.2594
49.0479
48.8365
48.4100
38.9148
26.6410
23.1055
17.8071
0.0000 TMS
f1 (ppm)

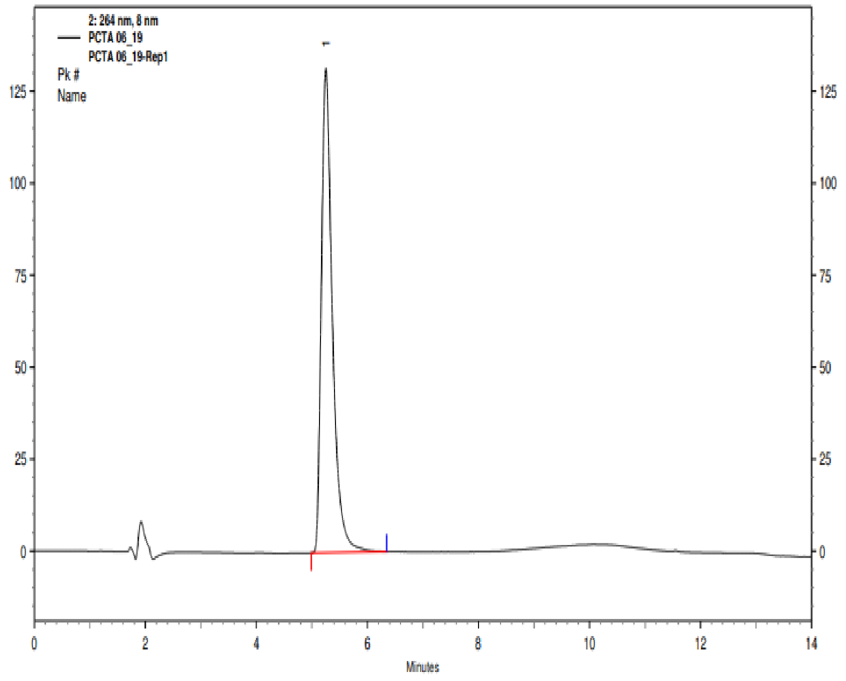
2: 264 nm, 8 nm
PCTA 06_19
PCTA 06_19-Rep1
Pk #
Name
Minutes

2: 264 nm, 8 nm

Pk #	Retention Time	Area	Area %	Height	Height %	Width
1	5.252	1834098	100.000	131796	100.000	1.36

N-(4-Methyl-3-((4-(pyridin-3-yl)pyrimidin-2-yl)amino)phenyl)-2-(4-(((tetrahydro-2H-pyran-2-yl)oxy)methyl)-1H-1,2,3-triazol-1-yl)acetamide (2g)

MW conditions: 180 min, 65 W, 70 °C (sealed tube). Yellow solid; yield: 42%, m.p.: 100-102 °C (95:5 DCM/MeOH). IR (cm^{-1}; film): 3266 (N-H str.); 1669 (C=O str.); 1117 and 1021 (C-O-C str.). ^{1}H NMR (MeOD, 400 MHz): 1.46-1.85 (m, 6H, CH_2), 2.29 (s, 3H, CH_3), 3.51-3.89 (m, 2H, CH_2), 4.64 (d, 1H, *J* = 12.4 Hz), 4.75 (t, 1H, *J* = 3.5 Hz, CH), 4.83 (d, 1H, *J* =12.5 Hz), 5.34 (s, 2H, CH_2), 7.22 (d, 2H, *J* = 1.8 Hz, Ar-H), 7.35 (d, 1H, *J* = 5.2 Hz, H-pyrimidine), 7.50 (dd, 1H, *J* = 7.9, 4.9 Hz, H-pyridine), 8.07 (s, 1H, H-triazole), 8.23 (s, 1H, Ar-H), 8.46 (d, 1H, *J* = 5.2 Hz, H-pyrimidine), 8.59 (dt, 1H, *J* = 8.0, 1.6 Hz, H-pyridine), 8.64 (m, 1H,H-pyridine), 9.25 (s, 1H, H-pyridine). ^{13}C NMR (MeOD, 101 MHz): 17.80, 20.35, 26.58, 31.58, 53.60, 61.04, 63.27, 99.39, 108.92, 116.76, 117.20, 125.64, 126.97, 128.54, 131.74, 134.20, 137.03, 137.62, 139.27, 146.02, 149.05, 151.86, 160.43, 162.49, 163.75, 165.70. HR-MS (ESI) *m/z* calculated for $C_{26}H_{28}N_8O_3Na$: 523.2182 $[M+Na]^+$; found: 523.2147 $[M+Na]^+$. HPLC-UV % (nm): 96.9 (264).

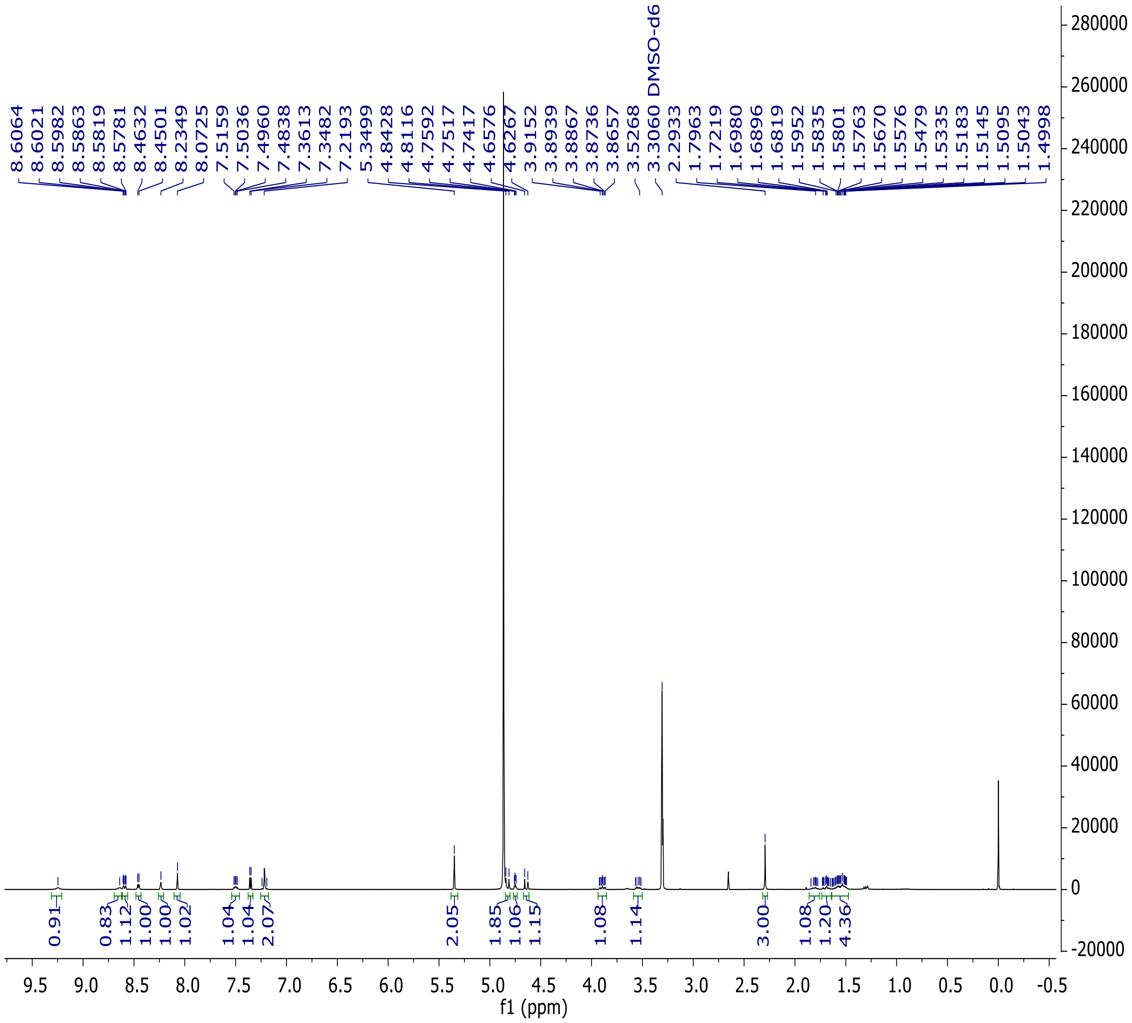

8.6064
8.6021
8.5982
8.5863
8.5819
8.5781
8.4632
8.4501
8.2349
8.0725
7.5159
7.5036
7.4960
7.4838
7.3613
7.3482
7.2193
5.3499
4.8428
4.8116
4.7592
4.7517
4.7417
4.6576
4.6267
3.9152
3.8939
3.8867
3.8736
3.8657
3.5268
3.3060 DMSO-d6
2.2933
1.7963
1.7219
1.6980
1.6896
1.6819
1.5952
1.5835
1.5801
1.5763
1.5670
1.5576
1.5479
1.5335
1.5183
1.5145
1.5095
1.5043
1.4998
0.91
0.83
1.12
1.00
1.00
1.02
1.04
1.04
2.07
2.05
1.85
1.06
1.15
1.08
1.14
3.00
1.08
1.20
4.36
f1 (ppm)

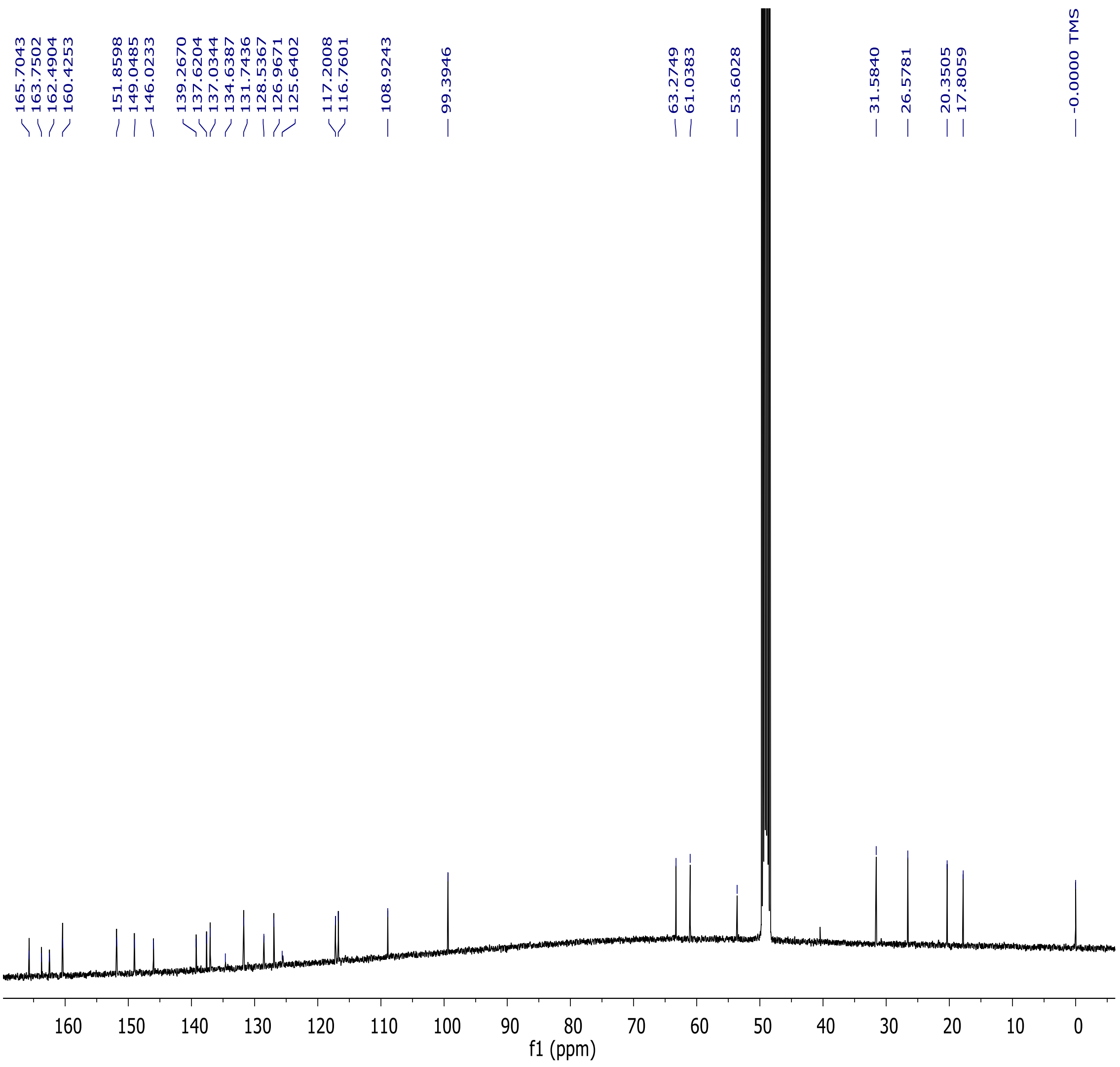
165.7043
163.7502
162.4904
160.4253
151.8598
149.0485
146.0233
139.2670
137.6204
137.0344
134.6387
131.7436
128.5367
126.9671
125.6402
117.2008
116.7601
108.9243
99.3946
63.2749
61.0383
53.6028
31.5840
26.5781
20.3505
17.8059
-0.0000 TMS
f1 (ppm)

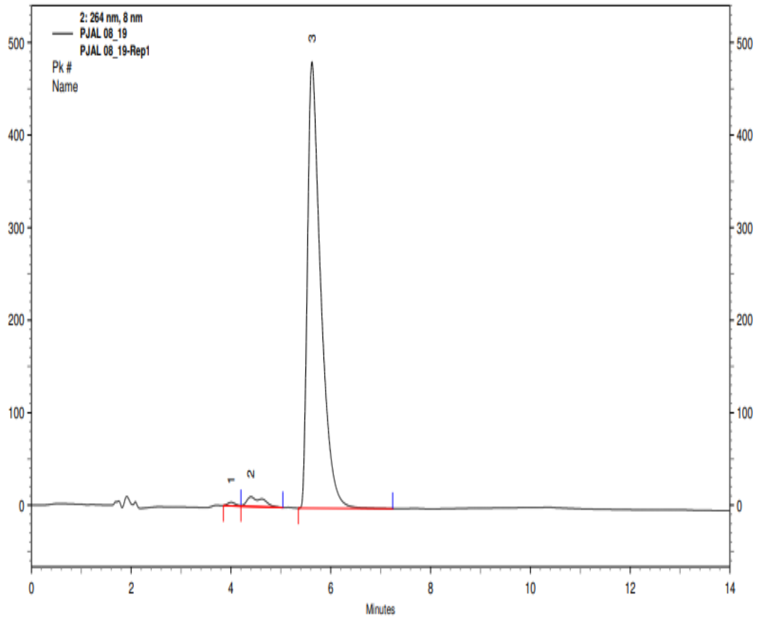
2: 264 nm, 8 nm
PJAL 08_19
PJAL 08_19-Rep1
Pk #
Name
Minutes

2: 264 nm, 8 nm

Pk #	Retention Time	Area	Area %	Height	Height %	Width
1	4.004	42688	0.466	3857	0.776	0.35
2	4.400	242771	2.650	10560	2.124	0.84
3	5.624	8877365	96.885	482782	97.100	1.89
Totals		9162824	100.000	497199	100.000	

_N_-(4-Methyl-3-((4-(pyridin-3-yl)pyrimidin-2-yl)amino)phenyl)-2-(4-phenyl-1_H_-1,2,3-triazol-1-yl)acetamide (2h)

MW conditions: 30 min, 100 W, 80 °C. Yellow solid; yield: 82%, m.p.: 229-231 °C. IR (cm^{-1}; film): 3433 (N-H str.); 1670 (C=O str.). ^{1}H NMR (DMSO-d_6, 400 MHz): 2.22 (s, 3H, CH_3), 5.39 (s, 2H, CH_2), 7.19 (d, 1H, J = 8.3 Hz, Ar-H), 7.28 (dd, 1H, J = 7.9, 1.6 Hz, Ar-H), 7.34 (t, 1H, J = 7.4 Hz, Ar-H), 7.40 – 7.50 (m, 3H, H-pyrimidine, Ar-H), 7.87 (d, 2H, J = 7.3 Hz, Ar-H), 8.01 (s, 1H, Ar-H), 8.46-8.57 (m, 2H, H-pyrimidine, H-pyridine), 8.61 (s, 1H, H-triazole), 8.92 (s, 1H, NH), 10.48 (s, 1H, NH). ^{13}C NMR (DMSO-d_6, 101 MHz): 17.51, 52.29, 107.73, 115.16, 115.46, 122.98, 125.02, 127.05, 127.75, 128.83, 130.27, 130.64, 133.98, 136.27, 137.93, 146.09, 148.21, 151.17, 159.35, 160.90, 161.85, 163.84. HR-MS (ESI) *m/z* calculated for $C_{26}H_{22}N_8ONa$: 485.1814 $[M+Na]^+$; found: 485.1789 $[M+Na]^+$. HPLC-UV % (nm): 97.9 (264).

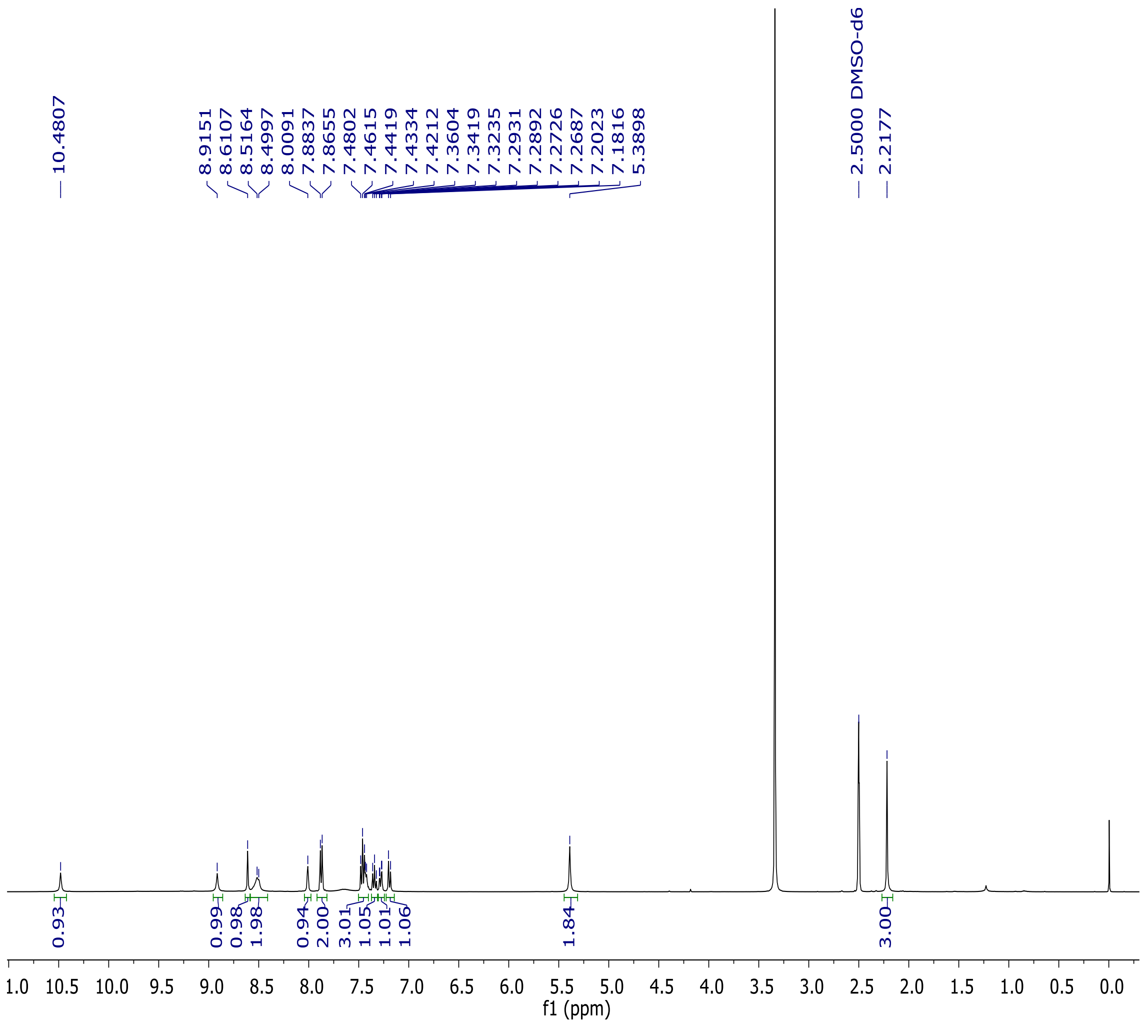
10.4807
8.9151
8.6107
8.5164
8.4997
8.0091
7.8837
7.8655
7.4802
7.4615
7.4419
7.4334
7.4212
7.3604
7.3419
7.3235
7.2931
7.2892
7.2726
7.2687
7.2023
7.1816
5.3898
2.5000 DMSO-d6
2.2177
0.93
0.99
0.98
1.98
0.94
2.00
3.01
1.05
1.01
1.06
1.84
3.00
f1 (ppm)

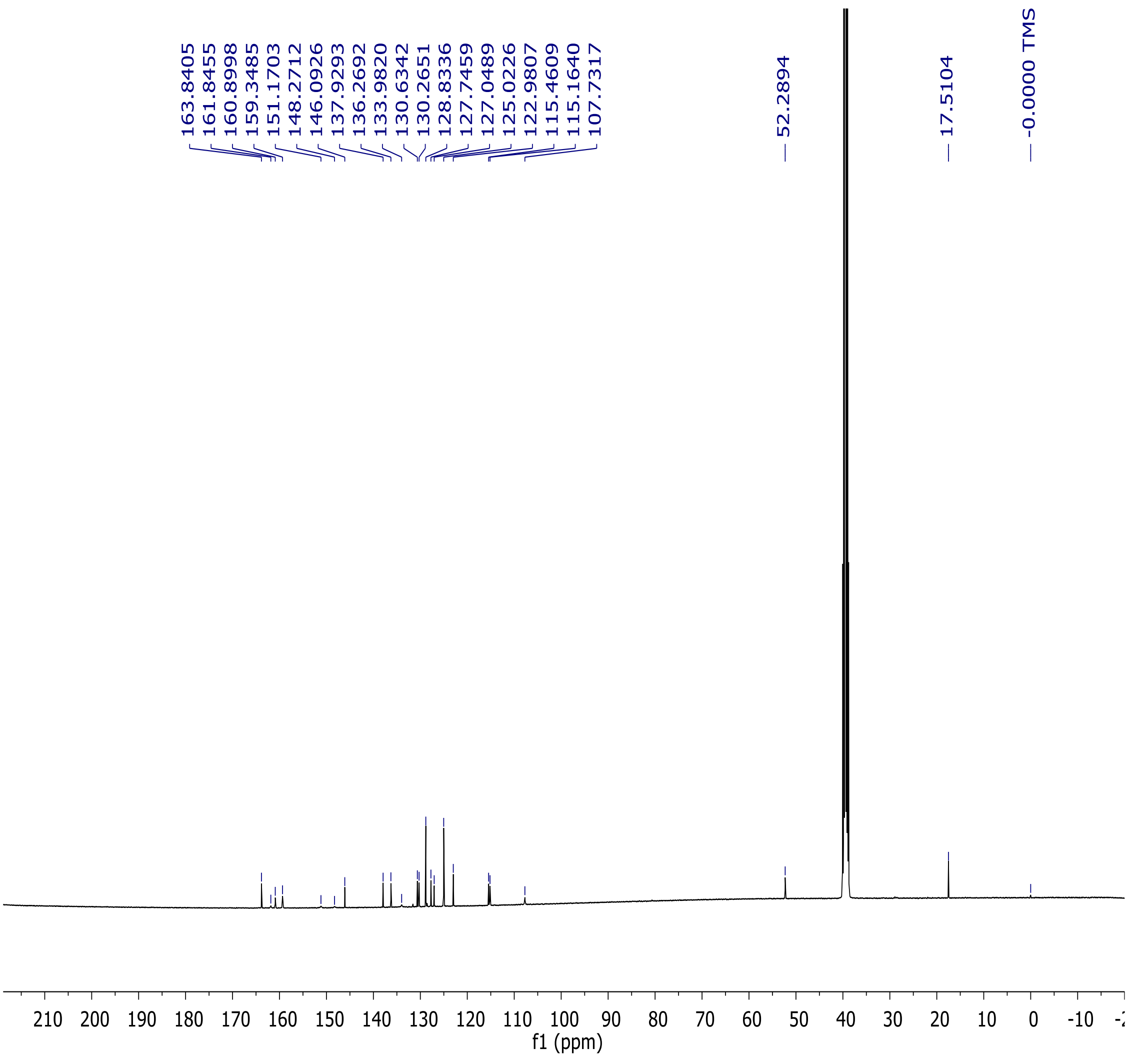
163.8405
161.8455
160.8998
159.3485
151.1703
148.2712
146.0926
137.9293
136.2692
133.9820
130.6342
130.2651
128.8336
127.7459
127.0489
125.0226
122.9807
115.4609
115.1640
107.7317
52.2894
17.5104
-0.0000 TMS
210 200 190 180 170 160 150 140 130 120 110 100 90 80 70 60 50 40 30 20 10 0 -10
f1 (ppm)

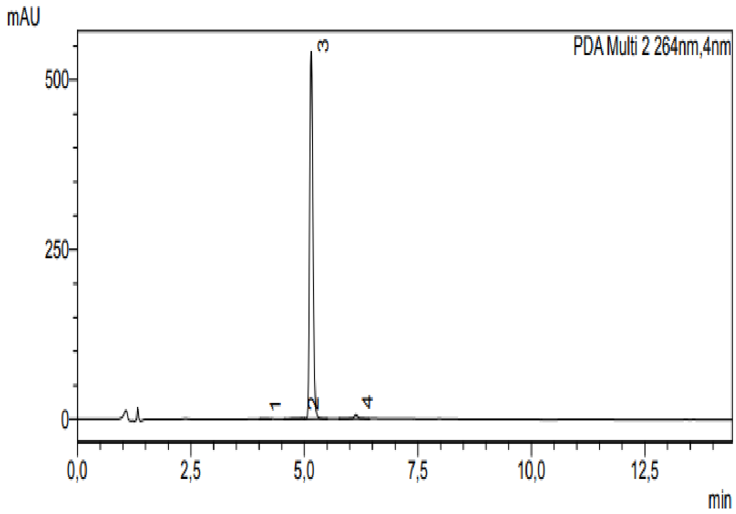
mAU
PDA Multi 2 264nm,4nm
500
250
0
1
2
3
4
0,0
2,5
5,0
7,5
10,0
12,5
min

PDA Ch2 264nm

Peak#	Ret. Time	Name	Area	Area%	Theoretical Plates/meter(USP)	Tailing Factor	Resolution(USP)	Capacity Factor(k')
1	4,11		3320	0,1	40905	1,147	--	--
2	4,94		20201	0,7	9800	--	2,296	0,203
3	5,15		2676925	97,9	122936	1,152	0,634	0,254
4	6,13		34021	1,2	146865	1,190	6,191	0,493
Total			2734467	100,0				

Methyl-1-(2-((4-methyl-3-((4-(pyridin-3-yl)pyrimidin-2-yl)amino)phenyl)amino)-2-oxoethyl)-1*H*-1,2,3-triazole-4-carboxylate (2i)

MW conditions: 15 min., 100 W, 80 °C. Brown solid; yield: 70%, m.p.:220-222 °C (95:5 DCM/MeOH). IR (cm^{-1}; film): 3256 (N-H str.); 1735 (C=Oesterstr.); 1681 (C=O amidestr.); 1204 and 1009 (C-(C=O)-O str.). ^{1}H NMR (MeOD, 400 MHz): 2.29 (s, 3H, CH_3), 3.93 (s, 3H, $COOCH_3$), 5.44 (s, 2H, CH_2), 7.21 (s, 2H, Ar-H), 7.35 (d, 1H, *J* = 5.2 Hz, H-pyrimidine), 7.50 (dd, 1H, *J* = 7.9, 4.9 Hz, H-pyridine), 8.25 (s, 1H, Ar-H), 8.46 (d, 1H, *J* = 5.2 Hz, H-pyrimidine), 8.55-8.60 (m, 1H, H-pyridine), 8.61-8.71 (m, 2H, H-triazole, H-pyridine), 9.24 (s, 1H, H-pyridine). ^{13}C NMR (MeOD, 101 MHz): 17.80, 52.63, 53.69, 108.95, 116.60, 117.10, 125.48, 128.44, 131.64, 131.75, 134.55, 137.00, 137.56, 139.27, 140.49, 149.07, 151.82, 160.42, 162.43, 162.52, 163.71, 165.19. HR-MS (ESI) *m/z* calculated for $C_{22}H_{20}N_8O_3Na$: 445.1737 $[M+Na]^+$; found: 445.1721 $[M+Na]^+$. HPLC-UV % (nm): 98.3 (264).

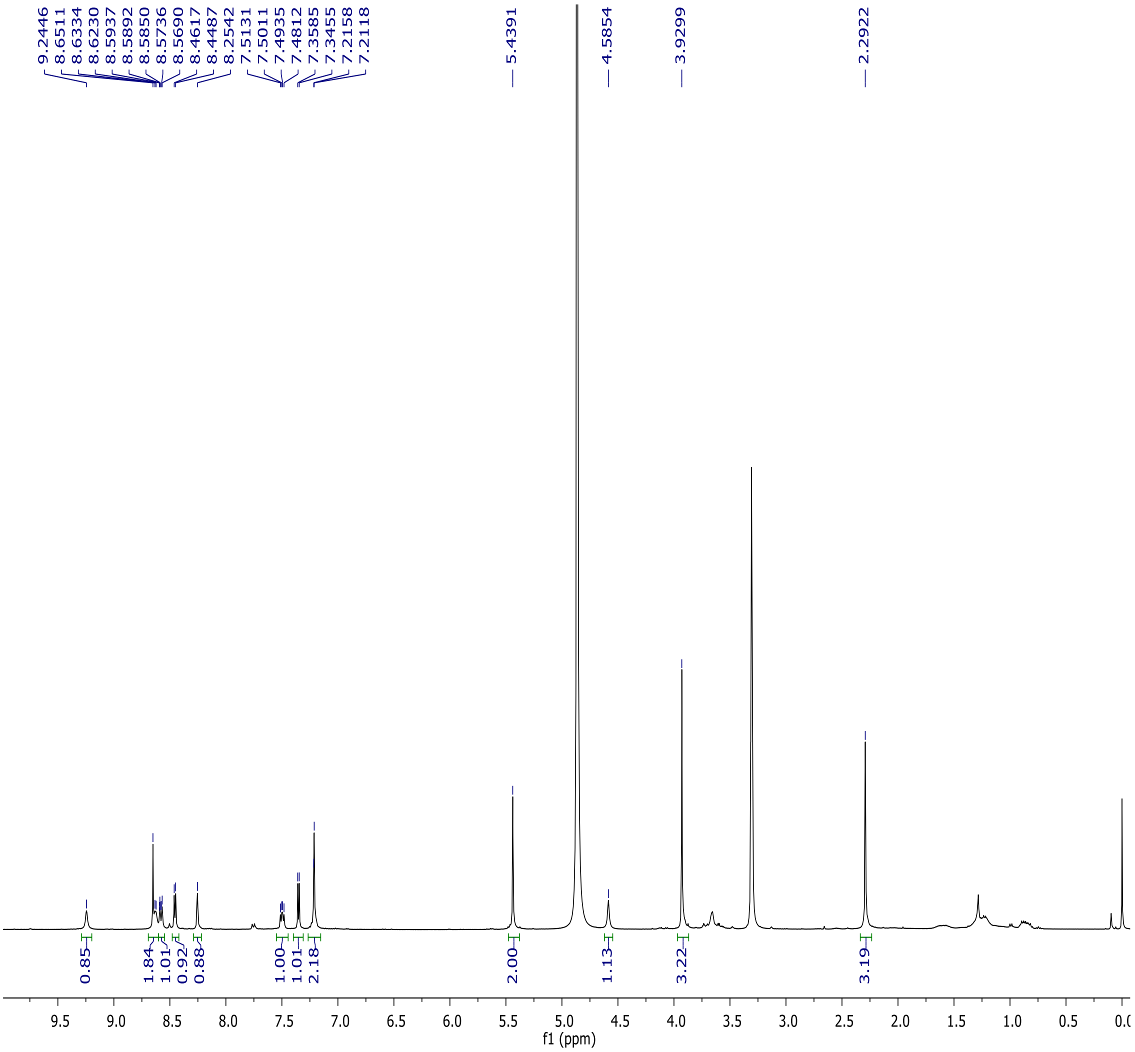
9.2446
8.6511
8.6334
8.6230
8.5937
8.5892
8.5850
8.5736
8.5690
8.4617
8.4487
8.2542
7.5131
7.5011
7.4935
7.4812
7.3585
7.3455
7.2158
7.2118
5.4391
4.5854
3.9299
2.2922
0.85
1.84
1.01
0.92
0.88
1.00
1.01
2.18
2.00
1.13
3.22
3.19
9.5 9.0 8.5 8.0 7.5 7.0 6.5 6.0 5.5 5.0 4.5 4.0 3.5 3.0 2.5 2.0 1.5 1.0 0.5
f1 (ppm)

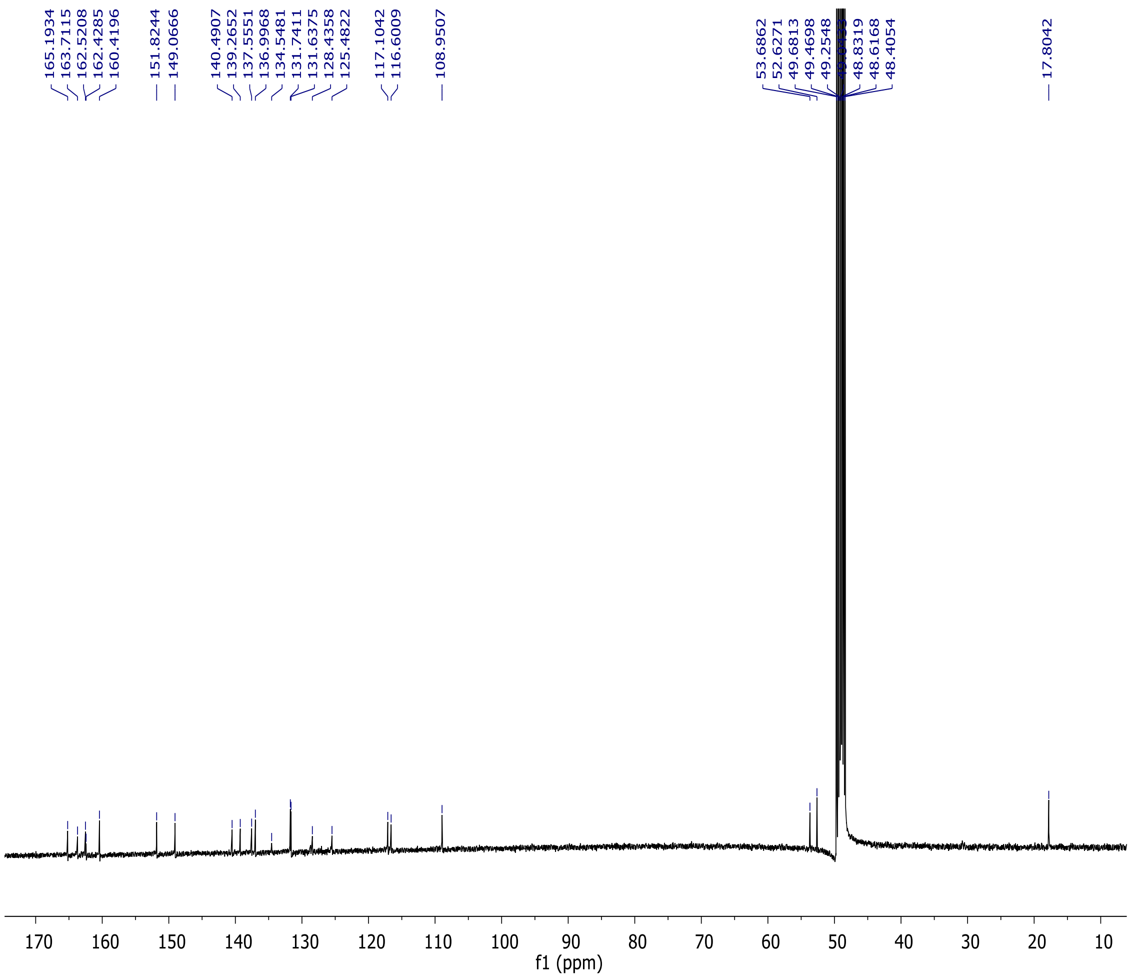

165.1934
163.7115
162.5208
162.4285
160.4196
151.8244
149.0666
140.4907
139.2652
137.5551
136.9968
134.5481
131.7411
131.6375
128.4358
125.4822
117.1042
116.6009
108.9507
53.6862
52.6271
49.6813
49.4698
49.2548
48.8319
48.6168
48.4054
17.8042
170 160 150 140 130 120 110 100 90 80 70 60 50 40 30 20 10
f1 (ppm)

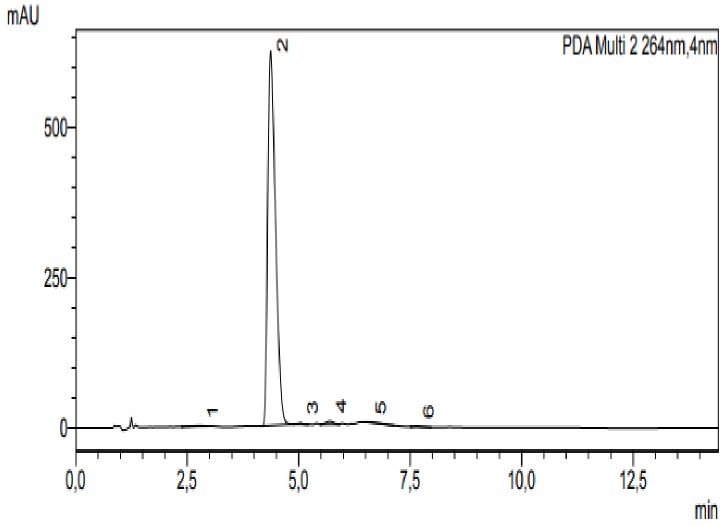

mAU
PDA Multi 2 264nm,4nm
500
250
0
1
2
3
4
5
6
0,0 2,5 5,0 7,5 10,0 12,5
min

PDA Ch2 264nm

Peak#	Ret. Time	Name	Area	Area%	Theoretical Plates/meter(USP)	Tailing Factor	Resolution(USP)	Capacity Factor(k')
1	2,81		13821	0,2	4730	0,640	--	--
2	4,37		7596704	98,3	18018	1,513	4,127	0,557
3	5,05		16704	0,2	89424	0,867	2,641	0,797
4	5,69		66034	0,9	28873	1,001	2,486	1,028
5	6,57		19949	0,3	17342	4,392	2,049	1,342
6	7,65		15264	0,2	64280	1,491	2,599	1,725
Total			7728477	100,0				

Ethyl-1-(2-((4-methyl-3-((4-(pyridin-3-yl)pyrimidin-2-yl)amino)phenyl)amino)-2-oxoethyl)-1*H*-1,2,3-triazole-4-carboxylate (2j)

MW conditions: 15 min, 100 W, 80 °C. Brown solid; yield: 80%, m.p.: 169-171 °C (95:5 DCM/MeOH). IR (cm^{-1}; film): 3271 (N-H str.); 1716 (C=O ester str.); 1663 (C=O amide str.); 1211 and 1061 (C-(C=O)-O str.). ^{1}H NMR (MeOD, 400 MHz): 1.38 (t, 3H, *J* = 7.1 Hz, CH_3), 2.29 (s, 3H, CH_3), 4.40 (q, 2H, CH_2), 5.43 (s, 2H, CH_2), 7.20 (d, 2H, *J* = 0.9 Hz, Ar-H), 7.34 (d, 1H, *J* = 5.2 Hz, H-pyrimidine), 7.48 (dd, 1H, *J* = 7.9, 4.9 Hz, H-pyridine), 8.26 (s, 1H, Ar-H), 8.45 (d, 1H, *J* = 5.2 Hz, H-pyrimidine), 8.57 (m, 1H, H-pyridine), 8.60-8.64 (m, 2H, H-triazole, H-pyridine), 9.24 (d, 1H, *J* = 1.8 Hz, H-pyridine). ^{13}C NMR (MeOD, 101 MHz): 14.61, 17.79, 53.66, 62.35, 108.91, 116.56, 117.06, 125.43, 128.40, 131.52, 131.70, 134.50, 136.96, 137.51, 139.23, 140.77, 149.02, 151.77, 160.37, 162.01, 162.39, 163.67, 165.15. HR-MS (ESI) *m/z* calculated for $C_{23}H_{22}N_8O_3Na$: 459.1893 $[M+Na]^+$; found: 459.1869 $[M+Na]^+$. HPLC-UV % (nm): 96.1 (264).

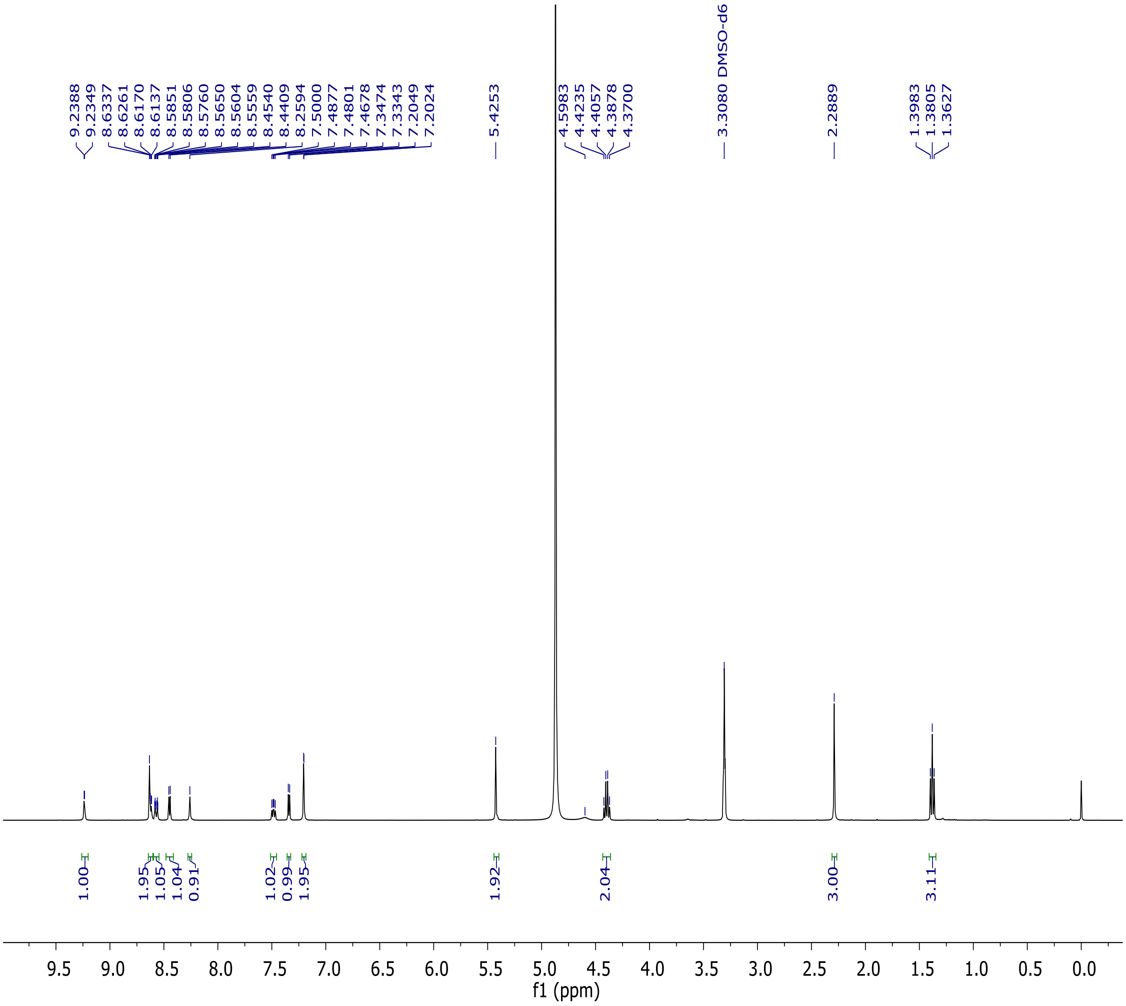
9.2388
9.2349
8.6337
8.6261
8.6170
8.6137
8.5851
8.5806
8.5760
8.5650
8.5604
8.5559
8.4540
8.4409
8.2594
7.5000
7.4877
7.4801
7.4678
7.3474
7.3343
7.2049
7.2024
5.4253
4.5983
4.4235
4.4057
4.3878
4.3700
3.3080 DMSO-d6
2.2889
1.3983
1.3805
1.3627
1.00
1.95
1.05
1.04
0.91
1.02
0.99
1.95
1.92
2.04
3.00
3.11
9.5 9.0 8.5 8.0 7.5 7.0 6.5 6.0 5.5 5.0 4.5 4.0 3.5 3.0 2.5 2.0 1.5 1.0 0.5 0.0
f1 (ppm)

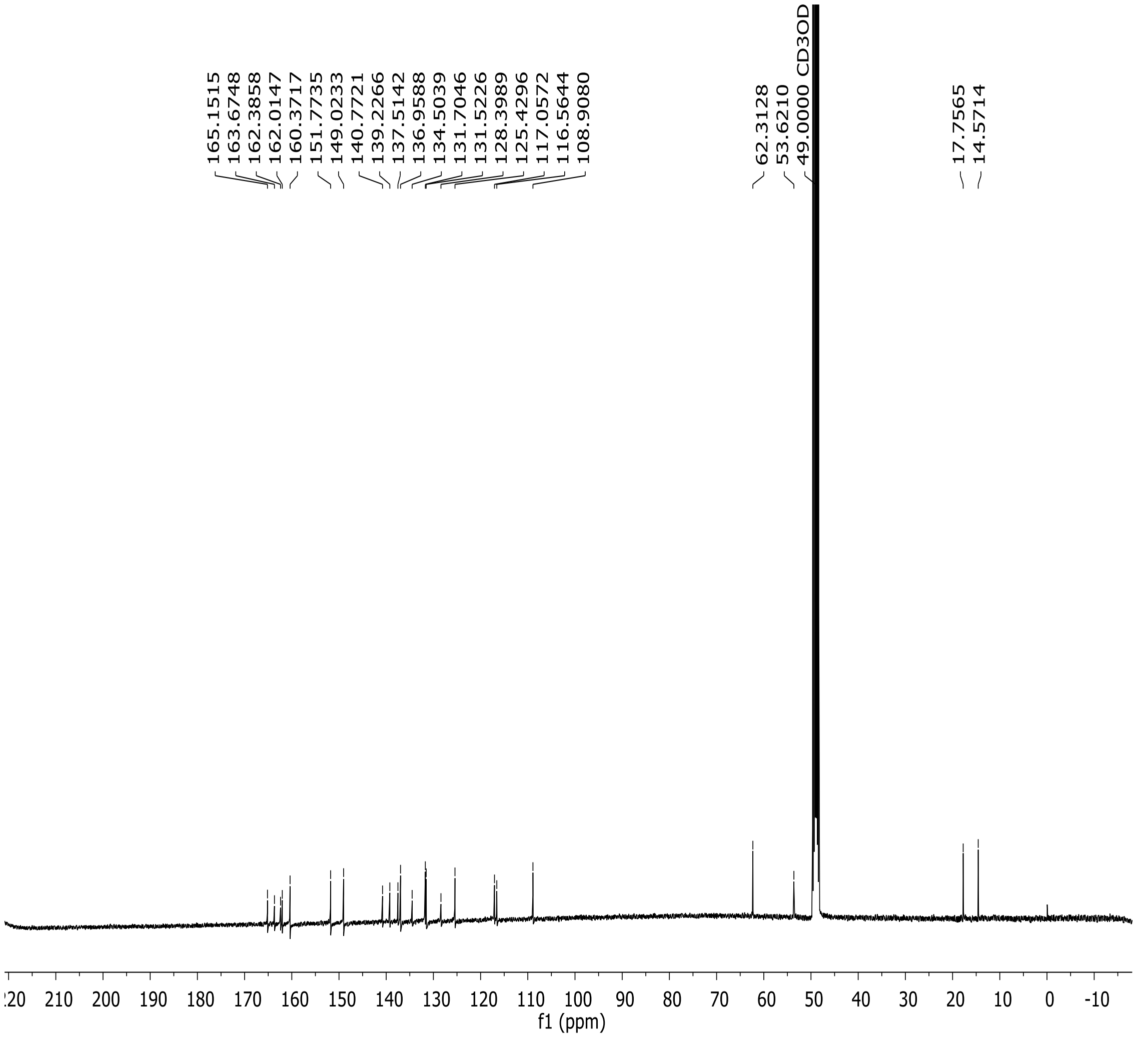
165.1515
163.6748
162.3858
162.0147
160.3717
151.7735
149.0233
140.7721
139.2266
137.5142
136.9588
134.5039
131.7046
131.5226
128.3989
125.4296
117.0572
116.5644
108.9080
62.3128
53.6210
49.0000 CD3OD
17.7565
14.5714
220 210 200 190 180 170 160 150 140 130 120 110 100 90 80 70 60 50 40 30 20 10 0 -10
f1 (ppm)

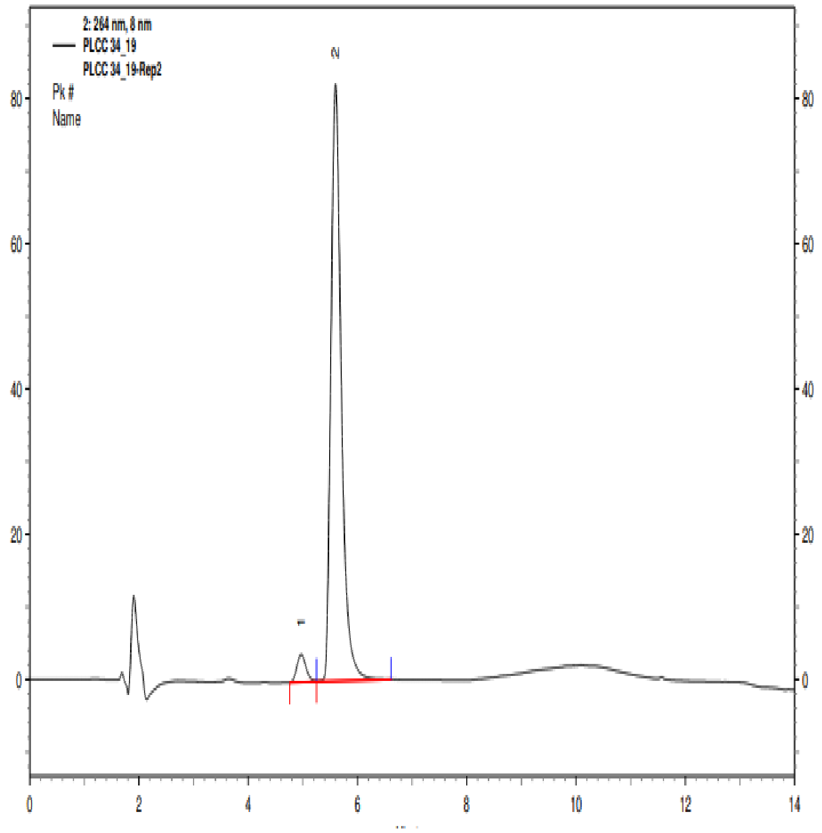

2: 264 nm, 8 nm

Pk #	Retention Time	Area	Area %	Height	Height %	Width
1	4.972	43981	3.894	3791	4.407	0.49
2	5.596	1085604	96.106	82234	95.593	1.36

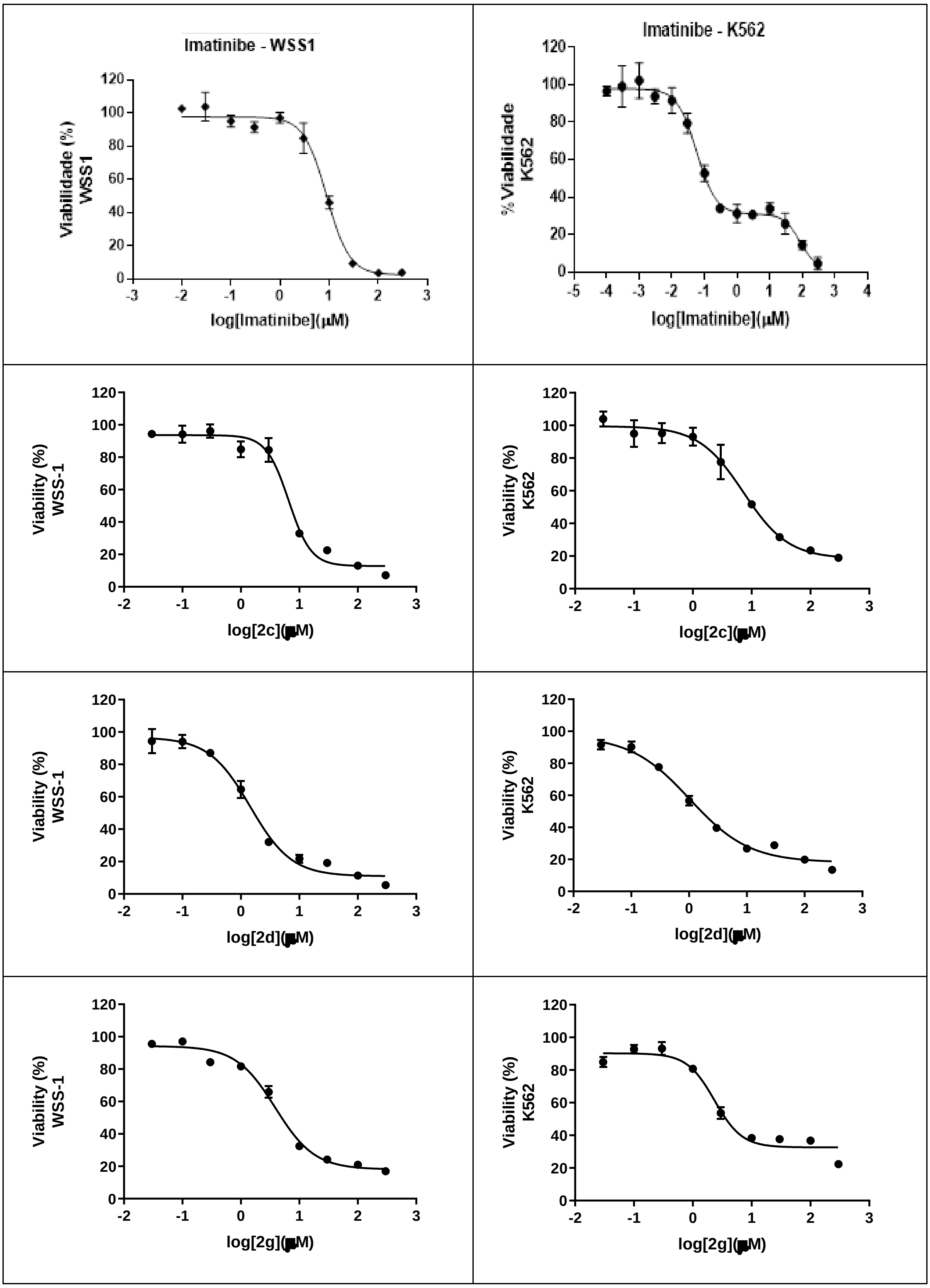
Imatinibe - WSS1
Viabilidade (%)
WSS1
log[Imatinibe](μM)
Imatinibe - K562
% Viabilidade
K562
log[Imatinibe](μM)
Viability (%)
WSS-1
log[2c](μM)
Viability (%)
K562
log[2c](μM)
Viability (%)
WSS-1
log[2d](μM)
Viability (%)
K562
log[2d](μM)
Viability (%)
WSS-1
log[2g](μM)
Viability (%)
K562
log[2g](μM)

References

1. Arioli, F.; Borrelli, S.; Colombo, F.; Falchi, F.; Filippi, I.; Crespan, E.; Naldini, G. A.; Scalia, G.; Silvani, A.; Maga, G.; Carraro, F.; Botta, M.; Passarella, D. ChemMedChem, 2011, 6(11), 2009-2018.
2. Genyi Meng, G.; Guo, T.; Ma, T.; Zhang, J.; Shen, Y.; Sharpless, K. B.; Dong, J. Nature, 2019, 574, 86–89.
3. Boechat, N.; Bastos, M. M.; Duarte, S. L.; Costa, J. C. S.; Mafra, J. C. M.; Daniel, L. C. C. *RVq* **2013**, *5*(2), 222-234.
4. Arioli, F.; Borrelli, S.; Colombo, F.; Falchi, F.; Filippi, I.; Crespan, E.; Naldini, G. A.; Scalia, G.; Silvani, A.; Maga, G.; Carraro, F.; Botta, M.; Passarella, D. ChemMedChem, 2011, 6(11), 2009-2018.
5. Moorhouse, A. D.; Moses, J. E. *Synlett* **2008**, *14*, 2089-2092.
6. Kim, D. Y.; Cho, D. J.; Lee, G. Y.; Kim, H. Y.; Woo, S. H.; Lee, H. E.; Kim, S. M.; Ahn, C. A. From Repub. Korean Kongkae Taeho Kongbo, KR 2012052095 A May 23, 2012.
7. Rao, Z.; Yang, C.; Chen, Y.; Bai, C.; Sun, T.; Pan, C.; Meng, F.; Li, Y.; Wang, J.; Jiang, Y. Tianjin International Joint Academy of Biomedicine, Peop. Rep. China, CN 106188005, Dec 07, 2016.
